# Autistic and non-autistic children’s perceptual decision-making in visual orientation and motion tasks and the effect of task instructions

**DOI:** 10.3758/s13414-026-03330-8

**Published:** 2026-09-06

**Authors:** Lou Thomas, Gaia Scerif, Hodo Yusuf, Mackenzie Laird, Grant Taylor, Nathan J. Evans, Catherine Manning

**Affiliations:** 1 School of Psychology and Clinical Language Sciences, University of Reading, Reading, UK; 2 Department of Experimental Psychology, University of Oxford, Oxford, UK; 3 Birkbeck, University of London, London, UK; 4 School of Psychology, University of Queensland, Brisbane, Australia; 5 Department of Psychology, University of Liverpool, Liverpool, UK; 6 School of Psychology, University of Birmingham, Birmingham, UK; 7 Centre for Developmental Science, University of Birmingham, Birmingham, UK

**Keywords:** Bayesian modeling, Decision making, Patient populations

## Abstract

**Abstract:**

Diffusion decision models (DDMs) offer the potential to go beyond standard accuracy and response-time indices to better understand perceptual decision-making in autism. One unanswered question is whether autistic participants can flexibly adjust the speed and accuracy of their decision-making according to task demands. Across two preregistered studies, 50 autistic and 50 non-autistic children aged 6–14 years completed a visual orientation task with no explicit instructions to be fast or accurate, and a visual motion task under both speed-emphasis and accuracy-emphasis instructions. These studies allowed us to investigate the influence of task, task instruction and modelling approach on group differences. We fit Bayesian hierarchical DDMs using a rigorous blind modelling approach, and follow-up two-step non-hierarchical analyses. Irrespective of task, instructions, and modelling approach, we found no conclusive evidence for group differences in DDM parameters. For the first time, we showed that autistic children can flexibly adjust their decision-making strategies according to speed–accuracy instructions. These results show that cognitive flexibility is not uniformly reduced in autism. To better understand within-participants variability, we investigated relationships between decision-making parameters and ADHD-related traits, reading ability, sensory processing and coordination skills. We found task-specific evidence for relationships between DDM parameters and sensory underresponsivity, sight-word reading and hyperactivity/impulsivity. Resolving inconsistent results when applying DDMs to autism will require contrasting modelling approaches, clear reporting of task instructions, and considering dimensions that co-occur with autism.

**Open practices statement:**

De-identified data are available in a safeguarded repository on the UK Data Service (https://doi.org/10.5255/UKDA-SN-858223). Experimental code, analysis scripts and output files are available for Study 1 (https://osf.io/vfqwx/) and Study 2 (https://osf.io/2gyc4/). Both Study 1 and Study 2 were preregistered (https://osf.io/gjksu, https://osf.io/2hdpg/). At the point of preregistration, we had collected data from one autistic participant, but we did not look at the data or complete any analyses before preregistration.

**Supplementary Information:**

The online version contains supplementary material available at https://doi.org/10.3758/s13414-026-03330-8.

## Introduction

Autism is a neurodevelopmental condition that influences how a person interacts with and experiences the world around them. Autistic people are all different, but there are some domains where autistic people commonly experience differences from non-autistic people. These include communication differences, and differences in sensory processing (ICD-11; World Health Organisation, 2019). In the latest revision to the ICD diagnostic criteria for autism, sensory processing differences are defined as “lifelong excessive and persistent hypersensitivity or hyposensitivity to sensory stimuli” (World Health Organisation, 2019). With an aim to understand how autistic people process sensory information, researchers have compared autistic and non-autistic people’s psychophysical thresholds in perceptual tasks. A mixed pattern of results has emerged (Simmons et al., 2009). Reduced thresholds have been reported for autistic people in some tasks, including orientation discrimination, which typically requires determining the tilt of gratings relative to a reference (Bertone et al., 2005; Dickinson et al., 2016; but cf. Shafai et al., 2015; Sysoeva et al., 2016). Yet increased thresholds have been reported for autistic people in other tasks, such as motion coherence, which requires determining the coherent direction of moving signal dots amongst randomly moving noise dots (Manning et al., 2013; Milne et al., 2002; Pellicano et al., 2005; Spencer et al., 2000, and see Van der Hallen et al., 2019, for review).

**Fig. 1:**
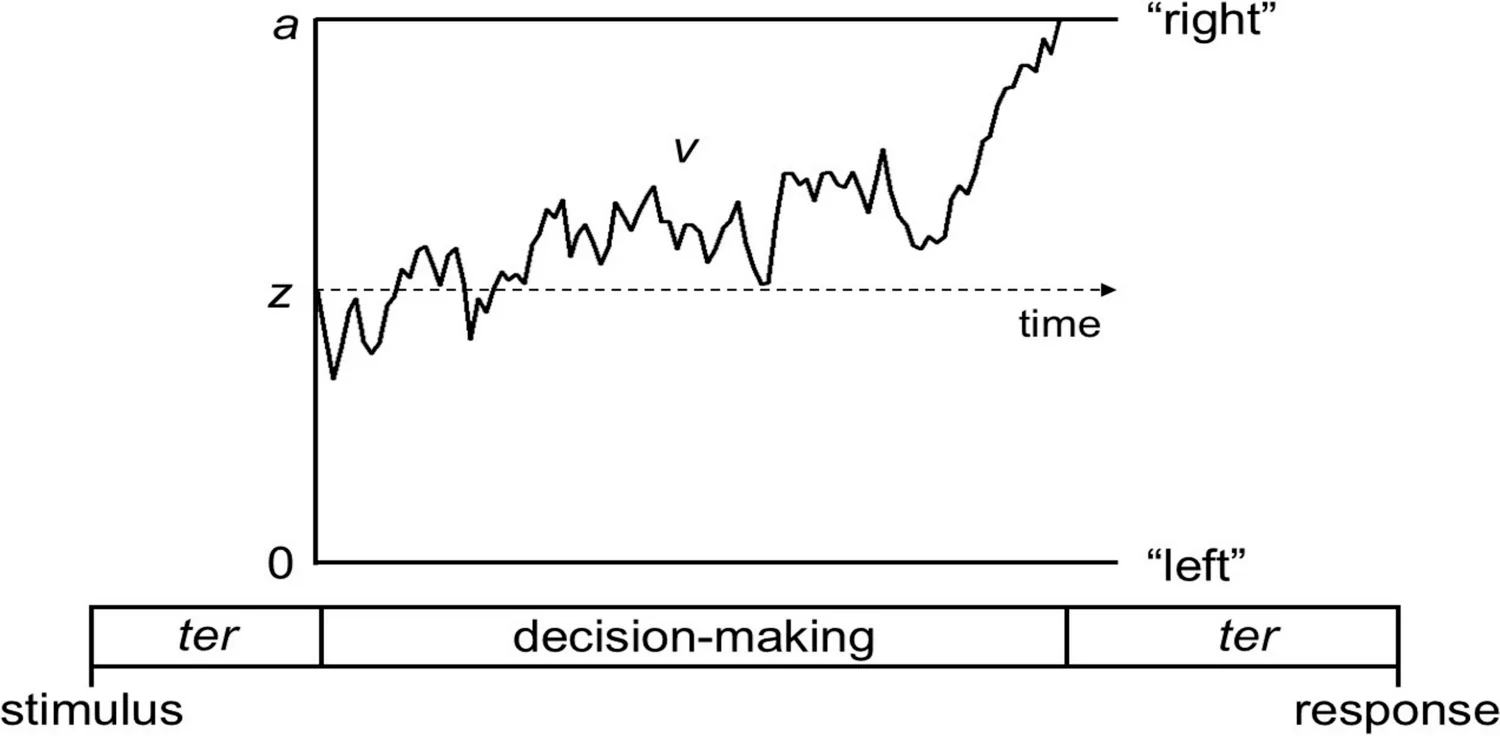
Schematic representation of decisional and non-decisional processes in diffusion decision models (DDMs). The decision-making process is modelled as a noisy evidence accumulation process from starting point, *z*, towards one of two decision bounds, corresponding to the response options. In Study 1, children judge whether the Gabor patch is oriented to the right or left of vertical, and in Study 2, children judge whether coherent motion is rightward or leftward. Drift rate, *v*, reflects the rate of evidence accumulation. Boundary separation, *a*, reflects response caution. Non-decision time, *ter*, is the time taken for sensory encoding and response generation. Figure reproduced from https://osf.io/rqw62/ under a CC-BY4.0 License

While psychophysical thresholds are based on the accuracy of responses in perceptual tasks, they neglect the multiple dynamic processing stages that contribute to performance. By contrast, diffusion decision models (DDMs) take both accuracy and response time distributions into account to model the timecourse from sensory encoding to response generation (Ratcliff & McKoon, 2008; Ratcliff & Rouder, 1998; White et al., 2010; see Fig. 1). This approach has the potential to tell us more about the reasons why autistic and non-autistic people differ in their perceptual performance in different tasks. Specifically, DDMs provide a theoretical framework to describe how noisy sensory evidence accumulates over time until it reaches one of two decision bounds (e.g., indicating clockwise versus anticlockwise orientation, or leftward versus rightward motion). The key parameters are drift rate (*v*), boundary separation (*a*), starting point (*z*), and non-decision time (*ter*). Drift rate reflects the efficiency of extracting task-relevant information: higher drift rates are typically observed on easier trials and in individuals with greater perceptual sensitivity. Boundary separation captures the amount of evidence a person requires before committing to a response. Wider boundaries indicate a preference for caution, whereas narrower boundaries indicate a willingness to respond with less accumulated evidence, and boundary settings can also reflect strategic adjustments to speed–accuracy trade-offs. The starting point represents any preexisting bias towards one response alternative. Non-decision time captures processes outside the decision itself, such as sensory encoding and motor execution, that contribute to the overall response time.

Decomposing decisions into distinct stages using DDMs can help us to understand the nature of group differences between autistic and non-autistic participants. For example, previous research applying DDMs to visual orientation task data has found that autistic participants responded more cautiously (i.e., wider boundary separation) than non-autistic participants in both adult (Pirrone et al., 2017) and child and adolescent (6–16 years; Pirrone et al., 2020) samples. For adults (Pirrone et al., 2017), the authors also found longer non-decision times, and no group differences in drift rate. The authors were unable to compare differences in drift rate in the child and adolescent sample (Pirrone et al., 2020), as stimulus difficulty was calibrated to each participant’s own performance. There were no differences in non-decision time. In both studies, the authors attributed longer response times in autistic participants compared with non-autistic participants to differences in response caution, instead of sensitivity to the stimulus (Pirrone et al., 2017, 2020). Without using the DDM framework, the authors may have concluded from the response time difference alone that autistic participants were less sensitive to the visual orientation stimuli than non-autistic participants. This therefore shows the potential of the DDM for helping better understand group differences between autistic and non-autistic participants.

The finding of wider boundary separation has also been replicated in studies applying DDMs to an implicit association task (Kirchner et al., 2012), performance on correct ‘go’ trials on a response inhibition go/no-go task (Karalunas et al., 2018), numerical addition problems (Iuculano et al., 2020) and a flanker task (Poole et al., 2024). These findings could be indicative of a more general cognitive preference for certainty in autistic people (Brosnan et al., 2014; Vella et al., 2018; Wilson & Bishop, 2022), and potentially linked to theories proposing that autistic and non-autistic people differ in how they process uncertainty (Van de Cruys et al., 2014). Specifically, highly precise prediction errors could lead to autistic people expecting a more precise signal when making decisions, which requires accumulating more sensory evidence (Hohwy & Palmer, 2014; Van de Cruys et al., 2014). However, other studies have found no evidence of wider boundary separation in autistic people. Manning et al. (2022a) reported no evidence for differences between autistic and non-autistic children’s response caution in a coherent motion task and another global motion task. Yusuf et al. (2025) similarly found no evidence for wider boundary separation in autistic adults compared with non-autistic adults in a numerosity task. There is even a report of *narrower* boundary separation from a study of autistic adolescents during face processing tasks (Powell et al., 2019). The presence and absence of differences in other DDM parameters (e.g., drift rate and non-decision time) across these studies is similarly mixed.

In this paper, we consider three possible reasons for these mixed results. First, group differences in DDM parameters may be task-dependent, reflecting different cognitive and neural processes. The Weak Central Coherence account (Happé & Frith, 2006) proposed reduced sensitivity to global characteristics of stimuli, whereas Enhanced Perceptual Functioning (Mottron et al., 2006) proposed heightened local perceptual functioning in autism. According to both theories, group differences may depend on the extent to which tasks engage local and global processing. Furthermore, the dorsal stream vulnerability account (Braddick et al., 2003) would predict group differences to be more pronounced for motion than form tasks, because of the greater demands on dorsal stream processes. Second, different modelling approaches could change the inferences made. Many of the previous studies reporting group differences used a two-step approach, first fitting a DDM to individual participants’ data and then conducting statistical comparisons between groups on the point estimates of DDM parameters for each participant (e.g., Pirrone et al., 2020). Meanwhile, Manning et al. (2022a) and Yusuf et al. (2025) used Bayesian hierarchical modelling and made inferences on the resulting posterior distributions, which takes account of uncertainty in individual participant-level parameters (see Boehm et al., 2018). Finally, differences in findings across studies may be, at least in part, due to differences in task instruction.

We know that non-autistic participants can flexibly modulate their decision-making strategies, showing wider boundaries when instructed to emphasise accuracy and narrower boundaries when instructed to emphasise speed (Mulder et al., 2010; Voss et al., 2004, 2019; Zhang & Rowe, 2014). However, no studies have investigated whether autistic participants can flexibly adjust their decision-making parameters according to speed–accuracy instructions. Following theoretical accounts of altered executive functioning in autism (Hughes et al., 1994; Ozonoff et al., 1994), it is conceivable that autistic participants adjust their decisional strategies less than non-autistic participants. Indeed, autism might overlap with ADHD, where reduced modulation of response caution has been reported (Mulder et al., 2010). On the other hand, it might be that autistic participants *can* update their performance based on explicit instructions, as shown in other paradigms (e.g., Helt et al., 2020; Plaisted et al., 1999; Senju et al., 2009), which could potentially explain previous inconsistent findings. In studies which have not reported group differences in response caution between autistic and non-autistic participants (Manning, Hassall, Hunt, Norcia, Wagenmakers, Evans et al., 2022a; Yusuf et al., 2025) participants were provided with explicit instructions to respond both quickly and accurately. However, to our knowledge, clear instructions were not given in some of the earlier studies reporting group differences in response caution (e.g., Pirrone et al., 2017). It is therefore possible that autistic participants are biased to responding cautiously but can modulate their boundary separation when instructed to respond quickly and accurately.

### Overview of studies

In the current paper, we collected data across two preregistered studies to help adjudicate between these different explanations for mixed results across previous DDM studies of autism, with a particular interest in autistic children’s ability to flexibly adjust their decision-making strategies. Autistic children and non-autistic children aged 6 to 14 years were presented with visual orientation (Study 1) and visual motion (Study 2) tasks. In Study 1, we investigated whether we could replicate the finding of wider boundary separation in autistic children for a visual orientation task (e.g., Pirrone et al., 2020), when no explicit instructions about speed and accuracy were given. We used a Bayesian hierarchical modelling approach as in previous work reporting null results (Manning et al., 2022a), although in supplementary analyses we checked whether the inferences changed with a two-step modelling approach. In Study 2, which was presented after Study 1, we determined whether autistic and non-autistic children’s task performance in a motion coherence task may be impacted by instruction and we explored group differences in the speed–accuracy trade-off. Children completed a motion coherence task twice, in a counterbalanced order: once with instructions to emphasise accuracy, and once to emphasise speed. While not previously used with autistic participants, this method has previously been used in a study exploring speed–accuracy trade-offs in children and adolescents with ADHD (Mulder et al., 2010). Our choice of tasks was theoretically motivated. While previous findings are mixed, *increased* sensitivity to orientation information has been reported in autistic people (Dickinson et al., 2016), potentially reflecting the local nature of the task (Mottron et al., 2006). Meanwhile, autistic people have been shown to have *reduced* sensitivity to motion coherence (Van der Hallen et al., 2019), which has been linked to the global nature of the task (Happé & Frith, 2006), and its reliance on the dorsal-stream (Braddick et al., 2003). Our use of these two tasks together was intended to highlight any task-dependence of group differences in DDM parameters.

As some evidence for a relationship between ADHD traits and perceptual decision-making parameters has been reported in children and adults (Manning, Hassall, Hunt, Norcia, Wagenmakers, Evans et al., 2022a; Mulder et al., 2010; Yusuf et al., 2025), we also investigated relationships between ADHD traits and model parameters, in both studies. We also conducted exploratory analyses to investigate relationships with other measures of individual differences (e.g., sensory processing, movement skills, and reading ability), to better understand the between-participants variability in DDM parameters reported in previous work (e.g., Manning et al., 2022a).

## Study 1: Orientation discrimination without explicit speed–accuracy instructions

### Preregistered hypotheses

In Study 1, we tested for group differences in DDM parameters for a visual orientation task without explicit speed–accuracy emphasis instructions. Our preregistered hypotheses (https://osf.io/gjksu) were as follows:

• Wider decision-bounds will be found in autistic participants relative to non-autistic participants, based on previous DDM studies with orientation tasks (Pirrone et al., 2017, 2020).
• No group difference will be found in drift rate (Pirrone et al., 2017, 2020).
• There will be either no group differences in non-decision times (Pirrone et al., 2020), or autistic participants will have longer non-decision times (Pirrone et al., 2017).
• It is also possible that group differences for parameters may be inconclusive, as in a previous study of autistic and non-autistic children’s motion processing, using the same Bayesian hierarchical DDM approach (Manning et al., 2022a).
• Drift rates will be negatively related to hyperactivity/impulsivity ADHD traits in the autism group (following Manning et al., 2022a).

### Methods

#### Participants

Our preregistered recruitment target was 50 autistic children with a confirmed autism diagnosis, and 60 non-autistic children with no diagnosed developmental conditions, allowing us to later select a matched comparison group (*n* = 50) based on age and performance IQ (PIQ). This sample size was based on previous related preregistered studies with a similar analysis method (Manning, Hassall, Hunt, Norcia, Wagenmakers, Evans et al., 2022a; Manning, Hassall, Hunt, Norcia, Wagenmakers, Snowling et al., 2022b). This sample is also larger than that previously included by Pirrone et al. (2017, 2020; i.e., 25 autistic and 32 neurotypical participants in the 2017 study, and a combined total of 29 participants in the 2020 study). Currently, there is no agreed method for conducting power analyses for Bayesian hierarchical DDMs. Our decision to include 50 participants per group was therefore deemed to be a minimum, following the inconclusive results obtained in Manning et al. (2022a; *n* = 50 per group), but with a higher number being practically infeasible due to the challenges associated with recruiting autistic participants within our age range.

Participants were recruited from schools, participant databases and community contacts. Our preregistered criteria led to the exclusion of a further 18 autistic and eight non-autistic participants. Of these, two autistic children and one non-autistic child had binocular Snellen visual acuities poorer than 6/6 (or poorer than 6/9 for children aged 6 to 8 years); seven autistic children and one non-autistic child had verbal and/or performance IQ scores below 70 (measured using the Wechsler Abbreviated Scales of Intelligence–Second Edition [WASI-II]; Wechsler, 2011); 4 autistic children scored below the cut-off for autism symptoms on both the Lifetime Social Communication Questionnaire (<15; Rutter et al., 2003) and the Autism Diagnostic Observation Schedule–Second Edition (Lord et al., 2012); two non-autistic children had Social Communication Questionnaire Scores above 15; five autistic children and one non-autistic child had incomplete datasets, having been unable to complete all tasks required for inclusion; and three children in the non-autistic group had a diagnosis or were awaiting an assessment for a developmental condition.

In addition to these preregistered exclusions, six further children were removed due to an error in the experimental code that caused all stimuli to be oriented leftwards, which was later rectified. For two autistic and two non-autistic children included in the final dataset, we were able to repeat the task with the correct code at a later date. While this meant that their data were collected after the motion coherence task (Study 2), transfer effects are unlikely due to the passage of time between sessions (>1 year).

After exclusions, 50 autistic and 60 non-autistic children met all inclusion criteria. We then selected the 50 non-autistic children who best matched the autistic group on age and PIQ using nearest-neighbour matching using the MatchIt package in R (Stuart et al., 2011; see also Manning et al., 2022a). Demographic characteristics for both groups are presented in Table 1. Autistic children were, on average, slightly older and had marginally lower IQ scores than non-autistic children. As age and IQ can affect diffusion model parameters (Lerche et al., 2020; Manning et al., 2021), we conducted additional analyses controlling for age and PIQ.

**Table 1:** Participant demographics in Study 1 and Study 2

	Study 1 (orientation)		Study 2 (motion)	
	Non-autistic	Autistic	Non-autistic	Autistic
**Gender (M:F)**	23:27	29:21	23:27	31:19
**Age**	10.64 (2.42) 6.37–14.85	11.18 (2.26) 6.00–14.70	10.62 (2.44) 6.37–14.63	11.19 (2.13) 6.00–14.70
**VIQ**	115.70 (12.57) 95–149	106.60 (13.80) 79–137	116.86 (13.51) 95–149	106.28 (14.30) 78–137
**PIQ**	114.10 (13.89) 85–142	108.88 (15.52) 82–140	114.52 (13.24) 87–142	109.16 (15.37) 82–140
**FSIQ**	116.62 (11.61) 90–144	108.64 (14.28) 80–135	117.48 (11.04) 90–144	108.60 (14.54) 80–135
**SCQ**	2.44 (3.02) 0–12	20.46 (7.37) 5–33	2.50 (2.84) 0–12	20.34 (7.40) 5–33
**Inattention**	-.89 (.87) -3.00–.44	1.00 (1.14) -2.22–3.00	-.82 (.87) -3.00–.50	.94 (1.10) -2.22–3.00
**Hyperactivity/impulsivity**	-.74 (1.00) -3.00–1.25	.99 (1.12) -3.00–2.67	-.71 (.97) -3.00–1.25	.97 (1.11) -3.00–2.67
**ADOS**	N/A	13.71 (4.41) 2–24	N/A	13.95 (4.35) 2–24
**Sensory OR**	2.98 (3.78) 0–16	20.54 (10.28) 1–39	3.18 (4.27) 0–18	20.04 (10.38) 1–39
**Sensory UR**	1.39 (2.60) 0–10	8.84 (4.80) 0–19	1.53 (2.77) 0–10	8.72 (4.79) 0–19
**TOWRE SWE**	108.67 (15.52) 74–145	95.00 (17.63) 58–134	109.47 (15.33) 74–145	95.22 (17.49) 58–134
**TOWRE PDE**	111.86 (12.75) 90–145	102.56 (14.74) 71–133	113.16 (12.92) 90–145	102.20 (14.88) 71–133
**DCDQ**	67.57 (8.89) 41–75	43.84 (14.93) 15–75	67.06 (9.11) 41–75	44.70 (15.69) 15–75

Apart from gender, descriptive statistics are presented as mean (*SD*) range. Inattention and hyperactivity/impulsivity were measured using the Strengths and Weakness of ADHD Symptoms and Normal Behavior Scale (SWAN; Swanson et al., 2012). VIQ = verbal IQ; PIQ = performance IQ; FSIQ = full-scale IQ; SCQ = Social Communication Questionnaire; ADOS = Autism Diagnostic Observation Schedule composite score (only collected with autistic children); Sensory OR = sensory overresponsivity; Sensory UR = sensory underresponsivity; TOWRE SWE = Test of Word Reading Efficiency Sight Word-Reading Efficiency; TOWRE PDE = Test of Word Reading Efficiency Phonemic Decoding Efficiency; DCDQ = Developmental Coordination Disorder Questionnaire

#### Apparatus

Stimuli were displayed on a Dell XPS 15 laptop (3,840 × 2,400 pixels) using MATLAB and PsychToolbox (Brainard, 1997; Pelli, 1997). Participants provided their responses using a Cedrus RB-540 Response Box.

#### Orientation task

The visual orientation task was presented as a game set in ‘InsectLand’. Participants were tasked with working out which way ‘striped beetles’ were facing (i.e., the orientation of single Gabor patches). Participants were told that there were two different types of striped beetle (with either stripes pointing to the left or right of vertical), and that they needed to help the zookeeper work out which way the stripes were facing so that he could get the beetles back into their correct boxes. In this task, participants were not given any instructions about completing the task quickly or accurately (no speed–accuracy emphasis).

**Fig. 2:**
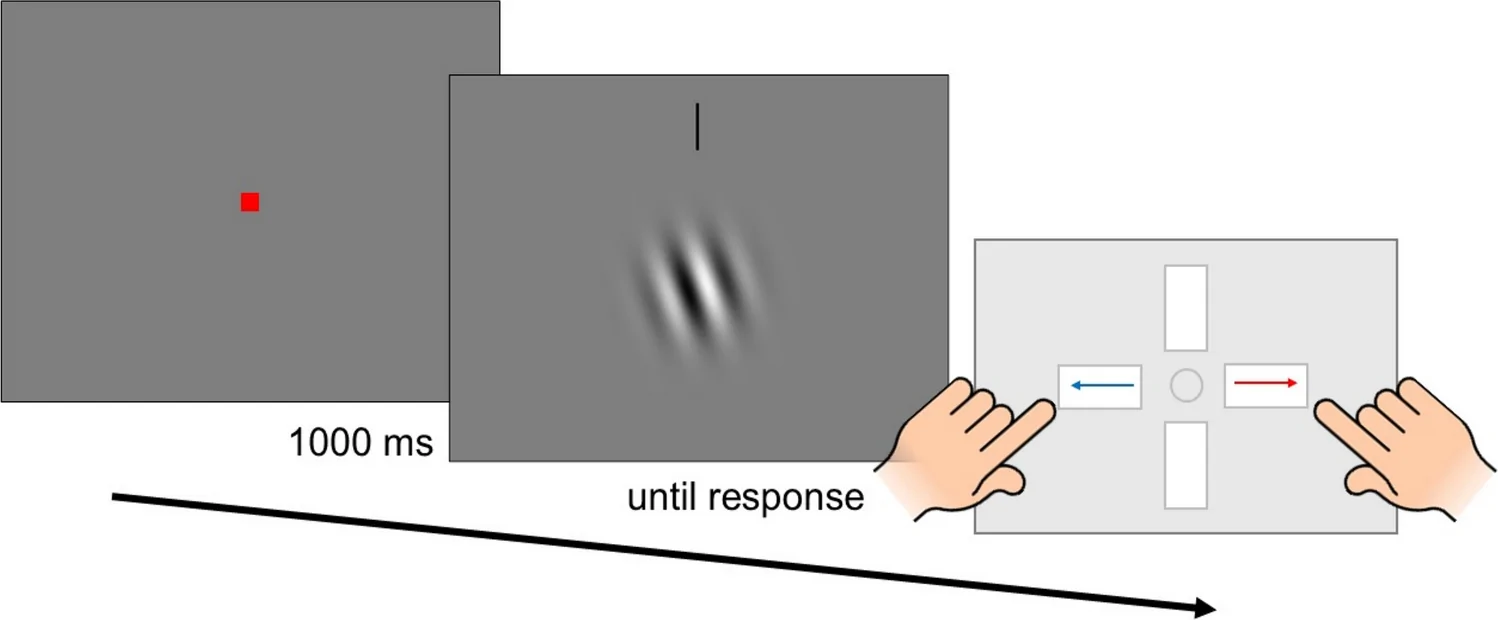
Schematic representation of stimulus in Study 1. A fixation square was presented for 1,000 ms, followed by a Gabor patch with a vertical reference line above it. Children were asked to determine whether the stripes were tilted to the left or right of the vertical line, and to respond with a button box. The stimulus depicted is a catch trial (−20° from vertical). (Colour figure online)

Each trial started with a fixation period of 1,000 ms, in which a red central fixation square (.29° wide) appeared on a grey background. Next, participants were shown a Gabor patch with a spatial frequency of .43 cycles per degree, presented centrally with a diameter of 11.72°, and a Gaussian envelope width of 1.68°. The stimulus was presented until the participant made a response (Fig. 2). The task procedure was explained verbally to participants, alongside an animation with task instructions on the screen. Participants were then presented with 5 blocks, or ‘levels’, the first of which was a practice block. During the practice block, children first completed six demonstration trials of increasing difficulty (+20°, −20°, +10°, −10°, −5°, +5°), so that the experimenter could explain the task. Red and blue boxes were displayed to the left and right of the stimulus, respectively, so that children could sort each ‘beetle’ into the correct box. Note that there were two more trials than stated in the preregistration, reflecting a typographical error. Following the demonstration trials, children were required to pass four consecutive trials in a criterion block of 20 trials, with easy (±5°) stimuli. In these trials and all future trials, the boxes on either side of the stimulus were not displayed and instead a black vertical line (2.18° in length) was presented above the stimulus to provide a reference point (as in Pirrone et al., 2020). Next, children completed eight practice trials, with mixed difficulty between ±5° and ±0.6°. Following each trial in the practice block, participants were told whether they responded correctly (i.e., “That was correct!” in green font, or “It was the other way that time” in black font).

The remaining blocks (Levels 2–5) each presented 38 trials in a random order. In each block there were 18 trials for each of two difficulty conditions: easy (±1.3° from vertical) and difficult (±0.6° from vertical), and two catch trials (±20° from vertical) to aid motivation. Half of the trials had a clockwise orientation and half had an anticlockwise orientation. The orientation levels were chosen with reference to the 65% and 85% correct thresholds reported for children aged 6 to 16 years by Pirrone et al. (2020), and refined through piloting (*n* = 10), to ensure that the task was possible for the youngest children, while ensuring sufficient errors for modelling. Participants made their response (i.e., left/anticlockwise, or right/clockwise orientation) using the first fingers of each hand positioned on the button box. In these blocks, children did not receive trial-by-trial feedback about their speed or accuracy. To aid motivation, points were given at the end of each block based on both speed and accuracy across the whole block (1/average response time × accuracy, rounded to the nearest integer, multiplied by 2). Children were always given at least 10 points, to maintain motivation. Participants were not informed how these points were calculated. There were opportunities for the children to take breaks in between blocks, and as needed. Experimental code is available online (https://osf.io/vfqwx/).

#### Standardised measures to investigate relationships with DDM parameters

Previous work (Manning, Hassall, Hunt, Norcia, Wagenmakers, Evans et al., 2022a) identified relationships between drift rate and the hyperactivity/impulsivity subscale of the Swanson, Nolan and Pelham (SNAP-IV) rating scale (Bussing et al., 2008; Swanson, 1992) in autistic children but not non-autistic children, which could have been due to a lack of variation in scores in the non-autistic children. In the current study, parents/carers completed the Strengths and Weaknesses of ADHD-symptoms and Normal-behaviour (SWAN) scale, which was designed to better capture continuous variation across the whole population (Swanson et al., 2012). The SWAN has been reported to have high internal consistency (Cronbach’s alpha >.9) and moderate test–retest reliability (Burton et al., 2019; Lakes et al., 2012). It is an 18-item scale where each item is rated on a 7-point scale from “far below average” (3) to “far above average” (−3). Nine items contribute to an inattention subscale, and nine items contribute to a hyperactivity/impulsivity subscale. We averaged responses for each subscale for each participant, where higher scores reflect greater difficulties (maximum = 3) and lower scores reflect greater strengths (minimum = −3).

We collected additional measures to better understand individual differences in children’s decision-making parameters. Parents/carers completed the sensory overresponsivity and sensory underresponsivity subscales of the Sensory Processing Scale Inventory Version 2.0 (Schoen et al., 2017), so that we could individually look at relationships with these distinct constructs (Manning et al., 2025). We summed the “yes” responses for each subscale (47 and 21 items for overresponsivity and underresponsivity, respectively), with higher scores reflecting more sensory features. These subscales have high internal consistency (Cronbach’s alpha >.88; Schoen et al., 2017).

Following previous work showing differences in drift rate in those with and without dyslexia (Manning, Hassall, Hunt, Norcia, Wagenmakers, Snowling, et al., 2022b) and between strong and poor readers more generally (O’Brien & Yeatman, 2021), we investigated relationships with reading ability. Children completed the Test of Word Reading Efficiency–Second Edition (TOWRE-2; Torgesen et al., 2012) and we extracted standardised scores for the Phonemic Decoding Efficiency and Sight Word Reading Efficiency subscales.

Finally, parents completed the Developmental Coordination Disorder Questionnaire (DCDQ; Wilson et al., 2009), as we reasoned that visual processing and response speed might be related to movement skills. This questionnaire is a 15-item measure that requires parents to compare their child’s coordination skills with other children of similar age, on a 5-point scale from “not at all” to “extremely” like their child. Item scores were summed to give a total score ranging from 15 to 75. The scale has high internal consistency (Cronbach’s alpha =.89; Wilson et al., 2009) and high test–retest reliability (>.90; Prado et al., 2009; Tseng et al., 2010).

#### Procedure

The visual orientation task was completed in a dimly lit room with the child sat at a viewing distance of 51 cm. A researcher carefully monitored the child throughout the task and provided regular task reminders and verbal encouragement. The orientation task took approximately 15 min to complete. Standardised measures were completed before or after the orientation task, with the exact order of standardised assessments varied across participants, to allow for differences in children’s attention. Children were given a record card on which they collected stamps as they progressed through the tasks. Parents/guardians of participants completed questionnaires whilst their children were taking part in the tasks. The study was given a favourable opinion for ethical conduct by the University of Reading Ethics Committee (ref: 21_37). All children were given a £10 voucher as a thank you for taking part in this Study and Study 2.

#### Modelling

The data were initially analysed by team members who were not involved in data collection and who were ‘blind’ to group membership (NJE and GT; see Dutilh et al., 2017). A dataset with group membership and ADHD traits randomly permuted was sent to the blind modellers, so that they could make all modelling decisions (e.g., excluding outliers, excluding participants whose performance suggests that they were not properly engaged in and/or understanding the task, choosing priors), without the potential for bias towards the hypotheses being tested that might come with deciding these criteria with unblinded data. The modelling code was then posted to the Open Science Framework before being run on the unblinded dataset by C.M.

At the blind modelling stage, two participants with very low accuracy were excluded from each group (leaving *n* = 48 per group). Specifically, their accuracy was below 80% in catch trials and their accuracy in the easy experimental trials was under 60%. Individual trials with response times <300 ms or >5,000 ms were excluded from analysis (6.58% of trials).

The basic model assessed group differences in DDM parameters. The model took trial-by-trial data as input and estimated five participant-level parameters (*v.mean*, *a*, *ter*, *v.diff*, *z*), with *v.diff* reflecting the difference in drift rate between difficulty levels (easy vs. difficult). There were hyper-parameters for the mean (µ) and standard deviation (σ) across groups, and the difference between groups (δ) for each parameter. The stochastic noise in the model (*s*) was fixed at 0.1, per convention. The priors for the basic model were:

- **Data level:**

$${y}_{pi} \sim diffusion\left({a}_{p, } {z}_{p},{Ter}_{p},{v}_{pi}, s\right)$$

- **Parameters:**

$$\begin{array}{lc}{a}_{p} \sim {N}_{+}\left({\upmu}_{a} \pm {\delta}_{a}, {\sigma}_{a}\right)\\ {z}_{p}/{a}_{p} \sim {TN}_{\mathrm{0,1}}\left({\upmu}_{z} \pm {\delta}_{z}, {\sigma}_{z}\right)\\ {Ter}_{p} \sim {N}_{+}\left({\upmu}_{Ter} \pm {\delta}_{Ter}, {\sigma}_{Ter}\right)\\ {v}_{p1}-{v}_{p2} \sim N\left({\upmu}_{v.diff} \pm {\delta}_{v.diff}, {\sigma}_{v.diff}\right)\\ \frac{{v}_{p1}+ {v}_{p2}}{2}\sim N\left({\upmu}_{v.mean} \pm {\delta}_{v.mean}, {\sigma}_{v.mean}\right)\\ s=0.1\end{array}$$

- **Hyperparameters:**

$$\begin{array}{lc}{\mu}_{a} \sim {N}_{+}\left(0.2, 0.2\right)\\ {\mu}_{z} \sim {TN}_{\mathrm{0,1}}\left(0.5, 0.2\right)\\ {\mu}_{Ter} \sim {N}_{+}\left(0.3, 0.3\right)\\ {\mu}_{v.diff} \sim N\left(0, 0.1\right)\\ {\mu}_{v.mean} \sim N\left(0.3, 0.3\right)\\ {\sigma}_{a},{\sigma}_{z},{\sigma}_{Ter},{\sigma}_{v.diff},{\sigma}_{v.mean} \sim \Gamma \left(1, 1\right)\\ {\delta}_{a},{\delta}_{z},{\delta}_{Ter},{\delta}_{v.diff},{\delta}_{v.mean} \sim N\left(0, 0.01\right),\end{array}$$

where *y* reflects the data, and subscripts* p* and *i* reflect the participant and difficulty level, respectively. The priors for the *µ* and *σ* parameters were based on those used in previous hierarchical DDM studies (Evans et al., 2019; Evans & Brown, 2017; Evans & Hawkins, 2019). The priors for the δ parameters were based on the “moderately informative priors” in Evans (2019). We used a differential evolution Markov chain Monte Carlo algorithm (DE-MCMC) to sample from the posterior with 3*k* interacting chains, where *k* is the number of individual-level parameters. Each chain had 4,000 iterations, with the first 1,500 discarded as burn-in. Chains were randomly migrated every 14 iterations between iterations 500 and 1,100. We assessed convergence using Gelman–Rubin diagnostic values, and ensured that all parameters underlying inferences (i.e. group-level parameters) were <1.1 (n.b. values were also <1.1 for almost all individual-level parameters).

To investigate relationships between DDM parameters and ADHD traits, we ran joint models where an ADHD score (inattention, hyperactivity/impulsivity) was covaried with *v.mean*, *a*, and *ter*, with additional hyper-parameters for each correlation, with bivariate normal priors.

- **Parameters:**

$$\begin{array}{lc}{{{a}}}_{{{p}}}\sim {{BN}}\left(\left[{{{\mu}}}_{{{a}}}\pm {{{\delta}}}_{{{a}}},{{{\mu}}}_{{{MOI}}}\pm {{{\delta}}}_{{{MOI}}}\right],\left[{{{\sigma}}}_{{{a}}}^{2},{{{\sigma}}}_{{{a}}}{{{\sigma}}}_{{{MOI}}}{{\rho}},{{{\sigma}}}_{{{MOI}}}{{{\sigma}}}_{{{a}}}{{\rho}},{{{\sigma}}}_{{{MOI}}}^{2}\right]\right)\\{{Ter}}_{{p}}\sim {{BN}}\left(\left[{{{\mu}}}_{{{Ter}}}\pm{{{\delta}}}_{{{Ter}}},\;{{{\mu}}}_{{{MOI}}}\pm{{{\delta}}}_{{{MOI}}}\right],\;\left[{{{\sigma}}}_{{{Ter}}}^{2},{{{\sigma}}}_{{{Ter}}}{{{\sigma}}}_{{{MOI}}}{{{\rho}}},{{{\sigma}}}_{{{MOI}}}{{{\sigma}}}_{{{Ter}}}{{{\rho}}},{{{\sigma}}}_{{{MOI}}}^{{{2}}}\;\right]\right)\\\left\lfloor\frac{{{{v}}}_{{{p1}}}+{{{v}}}_{{{p2}}}}{{{2}}},{{{MOI}}}\right\rfloor{{{\sim}}}\\{{BN}}\left(\left[{{{\mu}}}_{{{v.mean}}}\pm{{{\delta}}}_{{{v.mean}}},{{{\mu}}}_{{{MOI}}}+{{{\delta}}}_{{{MOI}}}\right],\;\left[{{{\sigma}}}_{{{v.mean}}}^{{{2}}},{{{\sigma}}}_{{{v.mean}}},{{{\sigma}}}_{{{MOI}}}{{{\rho}}},{{{\sigma}}}_{{{MOI}}}{{{\sigma}}}_{{{v.mean}}}{{{\rho}}},{{{\sigma}}}_{{{MOI}}}^{{{2}}}\right]\right)\end{array}$$

where *MOI* reflects the “measure of interest” (e.g., each ADHD score).

To establish the robustness of our results, we also ran variants of the models which, a) estimated between-trial variability in drift rate, *sv*, b) included a uniform contaminant process, and c) partialled out age and performance IQ. The priors at the “parameter” level for *sv* were the same as for *v.mean*, *a*, and *ter*. The priors for the hyperparameters of *sv* were:

$$\begin{array}{l}{\mu}_{sv}\sim N(\mathrm{0.1,0.1})\\ {\sigma}_{sv}\sim \Gamma \left(\mathrm{1,1}\right),\end{array}$$

For the contaminant process, the probability density function used to fit the model was a mixture of the standard DDM and a model of data contamination (Ratcliff & Tuerlinckx, 2002). Our model of data contamination assumed that contaminant responses were random in terms of timing and choice, meaning that it was a uniform distribution between our minimum and maximum allowed response times, equally split over the two response alternatives. This resulted in one additional individual-level parameter in the model, *contam*, which was the probability of contamination in the mixture. Like *sv*, we also related *contam* to the measures of interest in the joint model (i.e., the priors at the “parameter” level were the same as for *v.mean*, *a*, and *ter*), though note that we did not include *sv* and *contam* together in any model. The priors for the hyperparameters of *contam* were:

$$\begin{array}{l}{\mu}_{contam}\sim N(\mathrm{0.05,0.05})\\ {\sigma}_{contam}\sim \Gamma \left(\mathrm{1,1}\right).\end{array}$$

To better understand heterogeneity across participants, we also ran further joint models to explore relationships between DDM parameters and other measures of individual differences (sensory processing scale inventory, TOWRE, DCDQ) using the same priors as above for ADHD traits.

We drew our inferences based on Savage–Dickey Bayes factors for the relevant group difference parameters. Bayes factors >1 suggest more evidence for the alternative hypothesis relative to the null hypothesis, whereas Bayes factors <1 suggest more evidence for the null hypothesis (i.e., no group differences). Following convention (Jeffreys, 1961; Lee & Wagenmakers, 2014), we interpret Bayes factors above 3 and under ⅓ to reflect ‘moderate’ evidence, and Bayes factors between ⅓ and 3 to reflect weak evidence, where conclusive inferences cannot be drawn.

### Results and discussion

#### Accuracy and response time

**Fig. 3:**
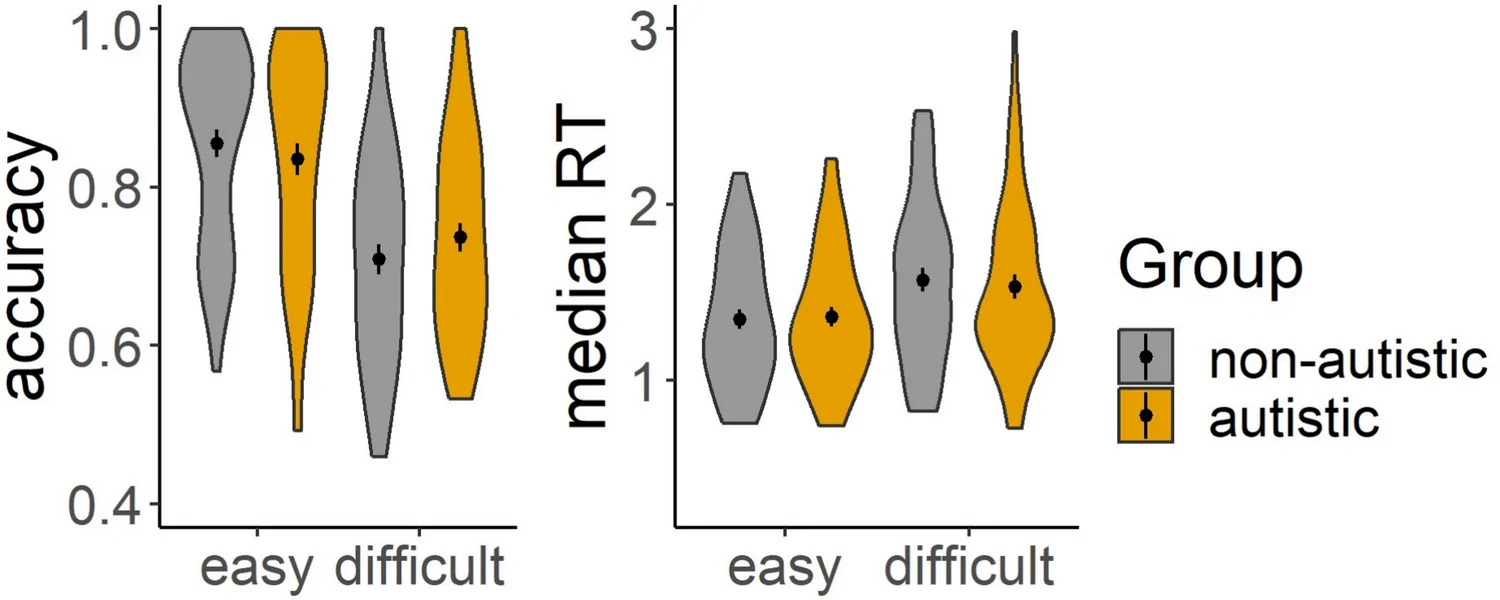
Violin plots of accuracy and median response time distributions for correct trials for each participant in the visual orientation task (Study 1). The grey and orange ‘violins’ reflect the kernel probability density of non-autistic and autistic children’s accuracy (proportion correct; left) and median RT in seconds for correct trials (right) for each difficulty level (easy: ±0.6°; difficult: ±1.3° from vertical). Median RTs are presented to minimise the influence of departures from normality in an individual’s RT data. Dots and vertical lines represent the group mean and ± 1 *SEM*

The mean accuracy and mean of the median response times for autistic and non-autistic children are shown in Fig. 3. We extracted median response times per condition for each participant to minimise the influence of departures from normality (Ratcliff, 1993). As expected, the difficult condition led to generally lower accuracies and longer median response times. While not a preregistered analysis relating to a hypothesis, a Bayesian factorial ANOVA in JASP (JASP Team, 2024) with default priors showed conclusive evidence of group differences in accuracy (BF_incl_ = 3.18) and an interaction between difficulty level and group (BF_incl_ = 12.74). Autistic children showed overall slightly higher accuracies than non-autistic children, but this was only true in the difficult condition (autistic: *M* =.74, *SD* =.12; non-autistic: *M* =.71, *SD* =.13). In the easy condition, this effect was reversed, with autistic children having slightly lower accuracies (*M* =.84, *SD* =.14) than non-autistic children (*M* =.86, *SD* =.12). However, for response times, there were no conclusive effects of group (BF_incl_ =.44) nor interactions with group BF_incl_ =.56) with relatively more evidence for the null hypothesis in both cases.

#### Preregistered analyses: Group differences in DDM parameters

**Fig. 4:**
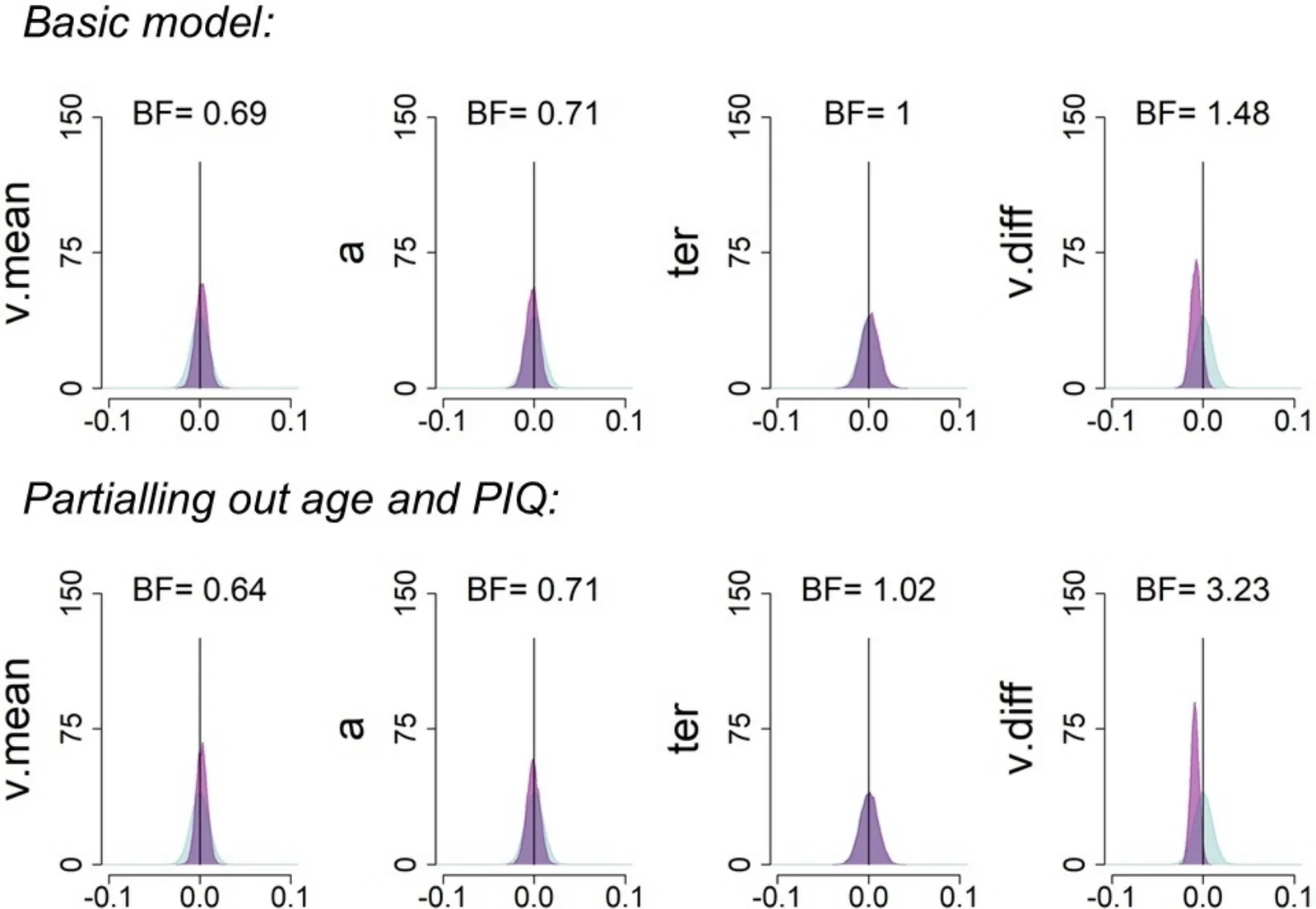
Prior (**blue**) and posterior (**purple**) density distributions for group differences in DDM parameters in Study 1. Posterior distributions shifted leftwards (negative) reflect lower values in the autistic group than the non-autistic group. The top row shows the distributions for the basic model without age and performance IQ (PIQ) partialled out. The bottom row shows the distributions for the model with age and performance IQ partialled out. Note: *v.mean* = mean drift rate across difficulty levels; *a* = boundary separation; *ter* = non-decision time; *v.diff* = difference in drift rate across difficulty levels. BF = Savage–Dickey Bayes factors, where BF > 1 reflects relatively more support for the alternative hypothesis over the null hypothesis. (Colour figure online)

In the basic DDM, conducted on the trial-by-trial data, all group differences were in the inconclusive range (see Fig. 4). There was relatively more evidence for the null hypothesis for group differences in boundary separation (BF = .71), and drift rate (BF = .69). There was equivocal evidence for the null and alternative hypotheses for group differences in non-decision time (BF = 1), and relatively more evidence for the alternative hypothesis for the difference in drift rates between easy and hard trials (*v.diff*, BF = 1.48). When partialling out age and performance IQ, the group difference in *v.diff* fell in the conclusive range (BF = 3.23, population effect size mean = −.46, 95% credible intervals [CI] = [−.91, −.01]), with the autistic children showing a smaller difference in drift rate between easy and hard trials than the non-autistic children. Specifically, autistic children had slightly lower drift rates in the easy condition and slightly higher drift rates in the difficult condition than non-autistic children, although these contrasts were inconclusive (BF =.83 and 1.46, respectively). There was no conclusive evidence for or against group differences in any parameters in the other model variants we tested, including those which modelled drift rate variability and those which modelled a contaminant process (see Supplementary Table 1).

#### Exploratory analyses: Group differences in DDM parameters with non-hierarchical modelling

As we did not find evidence for group differences in our preregistered analyses (cf. Pirrone et al., 2020), we considered whether using a two-step approach, by running a non-hierarchical DDM fit and conducting statistical tests on extracted point estimates, would influence our inferences. Following Pirrone et al. (2020), we ran a Bayesian ANCOVA on each individual’s posterior mean drift rate (in JASP, with default priors), with group as a between-participants factor, difficulty level as a within-participants factor, and performance IQ as a covariate. The best model of drift rate in terms of the marginal likelihood included difficulty and performance IQ, suggesting no effect of group (BF_incl_ = .47) nor interaction between group and difficulty level (BF_incl_ = .72). Bayesian ANCOVA on boundary separation and non-decision time with performance IQ as a covariate also showed no effect of group (BF_incl_ = .23 and .27, respectively). This confirms that regardless of the modelling approach chosen, there is no evidence in support of group differences in overall drift rate, boundary separation or non-decision time. However, we note that in the case of boundary separation and non-decision time, this inference was made more conclusively (BF < 1/3) when conducting statistical analyses on individual point estimates.

#### Preregistered analyses: Relationships between DDM parameters and ADHD traits

Next, we investigated relationships between DDM parameters and Inattention and Hyperactivity/Impulsivity subscales, using joint models. Although our preregistered hypothesis concerned the autistic group specifically, we first present the results across the whole sample, before evaluating relationships within each group separately. This order reflects the higher statistical power of the whole-sample model. Across the whole sample, there was no conclusive evidence for relationships between hyperactivity/impulsivity and DDM parameters. Specifically, there was conclusive evidence *against* relationships between hyperactivity/impulsivity and mean drift rate (posterior mean correlation = −.13, 95% CI [−.33, .07], BF = .29), and between hyperactivity/impulsivity and boundary separation (posterior mean correlation = −.05, 95% CI [−.25, .16], BF = .15), with inconclusive evidence (but with relatively more evidence for the null hypothesis) for a relationship between hyperactivity/impulsivity and non-decision time (posterior mean correlation = −.17, 95% CI [−.36, .03], BF = .54). Similarly, there was no conclusive evidence for relationships between inattention and DDM parameters across the whole sample (drift rate: posterior mean correlation = −.09, 95% CI [−.29, .12], BF = .19; boundary separation: posterior mean correlation = −.17, 95% CI [−.36, .04], BF = .51; non-decision time: posterior mean correlation = −.20, 95% CI [−.39, .00], BF = .81). Indeed, there was no conclusive evidence for or against relationships between inattention or hyperactivity/impulsivity and DDM parameters in any of the model variants we tested (Supplementary Table 2).

Similarly, no conclusive evidence for or against relationships was found in either group separately (Supplementary Table 3). In contrast to our hypothesis, there was conclusive evidence against the relationship between hyperactivity/impulsivity and drift rate in the autistic group (posterior mean correlation = −.06, 95% CI [−.31, .19], BF = .19). There was relatively more evidence for a negative relationship between inattention and non-decision time in the autistic group (posterior mean correlation = −.26, 95% CI [−.49, .00], BF = 1.23), but this was inconclusive. All other Bayes factors were under 1.

#### Exploratory analyses: Relationships between DDM parameters and other individual differences measures

We conducted exploratory analyses to investigate relationships between DDM parameters and coordination skills (DCDQ), sensory processing (SPSI) and reading ability (TOWRE). In the basic model, there was conclusive evidence against relationships between DCDQ scores and DDM parameters (BF ≤.15, Supplementary Table 4). However, when controlling for age and PIQ, there was relatively more evidence for a positive relationship between DCDQ score and non-decision time (posterior mean correlation = .23, 95% CI [.03, .41], although inconclusive (BF = 1.58; Supplementary Table 5). The relationship between DCDQ and boundary separation was also inconclusive, with relatively more evidence for the null hypothesis of no relationship (BF = .36). In the basic model, there was conclusive evidence against relationships between all DDM parameters and sensory processing scores (Supplementary Table 4) apart from the relationship between sensory underresponsivity and boundary separation, which was inconclusive (posterior mean correlation = −.21 [−.40, −.01], BF = 1.06). However, a different pattern emerged when controlling age and PIQ. Specifically, sensory underresponsivity was conclusively, negatively related to non-decision time (posterior mean correlation = −.28, 95% CI [−.45, −.08], BF = 6.15), and inconclusively, positively related to drift rate (posterior mean correlation =.25, 95% CI [.04, .43], BF = 1.98; Supplementary Table 5). In the basic model, there was no conclusive evidence for or against relationships between TOWRE SWE or PDE and DDM parameters. However, there was relatively more evidence for the alternative hypothesis between TOWRE SWE and boundary separation (posterior mean correlation = −.24, 95% CI [−.43, −.03], BF = 1.70). This relationship was not apparent when controlling for age and performance IQ (Supplementary Table 5). In fact, there was no conclusive evidence for or against any relationships between TOWRE scores and DDM parameters when partialling age and PIQ.

### Discussion of Study 1 results

Based on previous work (Pirrone et al., 2017, 2020), we hypothesised that autistic children would show wider decision-bounds than non-autistic children, no group differences in drift rate, and either no group differences in non-decision time, or longer non-decision times. However, we found inconclusive evidence for or against all group differences. We had also foreseen the possibility that the results might be inconclusive, following previous work with this Bayesian hierarchical DDM approach and a similarly selected sample of children (Manning, Hassall, Hunt, Norcia, Wagenmakers, Evans et al., 2022a). To ascertain whether the inconclusive results were attributable to our modelling approach, we conducted a two-step analysis more similar to Pirrone et al. (2020). We fit the DDM to each individual participant’s dataset, extracted posterior mean estimates for each parameter, and conducted Bayesian ANCOVAs. Here we found inconclusive evidence for group differences in drift rate, but conclusive evidence *against* group differences in boundary separation and non-decision time. Therefore, this difference in analytical approach affected the level of conclusiveness reached, but importantly, the results are still at odds with Pirrone et al. (2020), who reported wider boundary separation in autistic children. Our study was not a direct replication of Pirrone et al. (2020), as they tailored the difficulty levels to each participant’s ability. Instead, we chose fixed difficulty levels close to the mean difficulty levels used in Pirrone et al., so that we could evaluate changes in drift rate. Another difference is that Pirrone et al. had almost twice as many trials as we did (280 trials, versus our 144 trials per participant), which would have enabled more precise estimates for each participant. However, we had more than twice as many participants as Pirrone et al. (*n* = 29), so that, overall, our dataset included many more trials. Another difference is the inclusion of our child-friendly game and block-level feedback (“points”) at the end of each block. This could have led to differences in participant motivation across the two studies. However, it is hard to determine how important these differences are.

The only indication of group differences in this study was unexpected, with a smaller difference in drift rates across the two difficulty levels between autistic and non-autistic children, when controlling for age and performance IQ. This is echoed by the accuracy results, where autistic children were more accurate in the difficult condition but less accurate in the easy condition, compared with non-autistic children. This result could reflect a differently shaped psychometric function in autistic and non-autistic children, whereby autistic children have overall shallower psychometric functions. However, this would need assessing in a study with more than two difficulty levels, to capture the entire psychometric function.

We also hypothesised that drift rates would be negatively related to hyperactivity/impulsivity traits in the autism group (following Manning, Hassall, Hunt, Norcia, Wagenmakers, Evans et al., 2022a), but here we found conclusive evidence against this relationship. This could result from differences in task (Manning et al. reported a relationship with motion processing tasks) or ADHD measure (here we used the SWAN, whereas Manning et al. used the SNAP-IV-c). However, we would have expected the SWAN to be more sensitive, as it is designed to capture continuous variation across typical and atypical development. In terms of the exploratory analyses, we found few conclusive relationships. The only conclusive relationship was a negative relationship between sensory underresponsivity and non-decision time, specifically when controlling for age and PIQ. This relationship is hard to interpret, as we would have expected participants with greater sensory underresponsivity to have longer non-decision times. It could be a spurious relationship, given that it only arose in the partial model, and the relationship will need to be replicated in future work. Ultimately, the fact that many results were inconclusive suggests large samples are likely needed to understand variability in children’s decision-making parameters.

## Study 2: Motion coherence discrimination with varying speed–accuracy instructions

### Preregistered hypotheses

In Study 2, we investigated group differences in DDM parameters for a motion coherence task, which allowed us to compare the pattern of results found in Study 1 for a different task which has typically been associated with reduced sensitivity in autistic populations (Van der Hallen et al., 2019). Crucially, the motion coherence task was presented under two conditions: once with instructions to emphasise speed, and once with instructions to emphasise accuracy. This allowed us to investigate whether autistic and non-autistic children differed in their ability to modulate the speed–accuracy trade-off following instructions.

Our preregistered hypotheses (https://osf.io/2hdpg/) were as follows:

- There may be differences in boundary separation (following Iuculano et al., 2020; Karalunas et al., 2018; Kirchner et al., 2012; Pirrone et al., 2017, 2020), particularly if task instructions are not important. However, inconclusive evidence for differences in DDM parameters may be found, following Manning, Hassall, Hunt, Norcia, Wagenmakers, Evans et al. (2022a).
- If autistic participants are able to successfully modulate their performance on the motion coherence task with instruction, there would be no group differences in the parameters that reflect group differences between the speed-emphasis and accuracy-emphasis conditions, with autistic participants showing equivalent ability to modulate their decision bounds. If autistic participants are less able to optimise the speed–accuracy trade-off (as in children with ADHD; Mulder et al., 2010), we expected to find a smaller difference in boundary separation between speed-emphasis and accuracy-emphasis conditions in autistic versus non-autistic children.
- Drift rates will be negatively related to hyperactivity/impulsivity traits in the autism group (as in Manning, Hassall, Hunt, Norcia, Wagenmakers, Evans et al., 2022a).
- The difference in boundary separation between speed-emphasis and accuracy-emphasis instructions will be negatively related to hyperactivity/impulsivity ADHD traits (following Mulder et al., 2010).

### Methods

The apparatus, standardised measures and overall procedure were as described in Study 1. Here we focus on points of difference from Study 1.

#### Participants

As in Study 1, our preregistered recruitment target was 50 autistic and 60 non-autistic children, following our preregistered exclusions, and later reduced to 50 participants in each group through best matching. The sample size justification was similar to that for Study 1, but additionally we ensured that it would allow us, in exploratory analyses, to detect the size of effects reported using a similar approach to Mulder et al.'s (2010) two-step analysis method. Mulder et al. (2010) obtained a difference in speed–accuracy trade-off between ADHD and non-ADHD participants with an effect size of *d* =.975. Using G*Power (Version 3.1.9.7; Faul et al., 2007), we concluded that 29 participants per group are required to detect this effect with 95% power in a two-tailed *t* test, so that our sample of 50 participants per group was powered to detect this difference.

Of the 100 participants included in the dataset for Study 2, 92 were also included in the dataset for Study 1. The same preregistered inclusion criteria were used as in Study 1, but we additionally excluded three non-autistic and two autistic children who could not complete both versions of the motion coherence task, and we again selected the 50 best matching non-autistic children for the 50 autistic children (see demographics in Table 1).

#### Motion coherence task

Participants completed two versions of a motion coherence task (based on Manning, Hassall, Hunt, Norcia, Wagenmakers, Evans et al., 2022a) which were presented as a child-friendly game set in ‘InsectLand’. Children were asked to help the zookeeper work out which way ‘fireflies’ were escaping out of a box. On one version of the task, children were asked to respond as accurately as possible (i.e., accuracy emphasis), and on the other version of the task, children were asked to respond as quickly as possible (i.e., speed emphasis). The order of versions was counterbalanced.

**Fig. 5:**
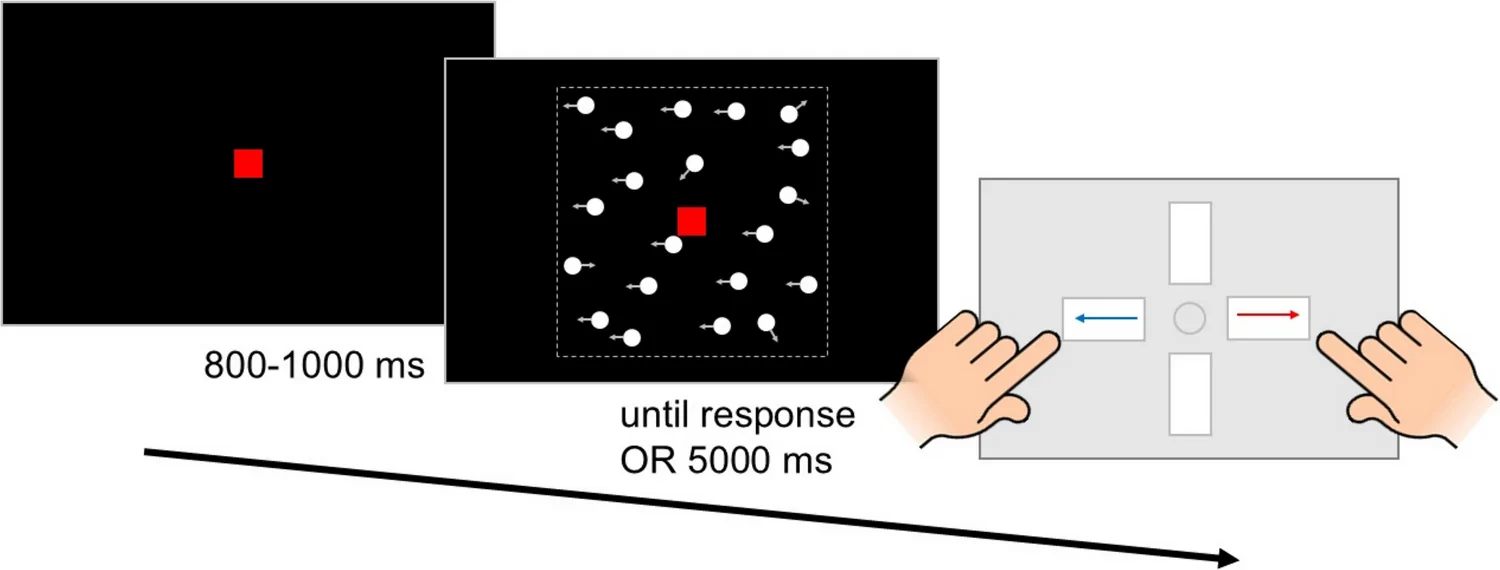
Schematic representation of stimulus in Study 2. A fixation square was presented for a randomly selected duration between 800 and 1,000 ms, followed by a set of white moving dots in which a proportion moved in a coherent direction. Children were asked to determine whether the coherent motion was leftwards or rightwards, and to respond with a button box. Note that dotted lines showing the stimulus aperture and arrows showing directions are drawn for illustrative purposes only. (Colour figure online)

Each trial started with a square fixation point (.25° width) presented for a randomly jittered duration of between 800 and 1,000 ms followed by the presentation of a set of 100 white dots (diameter .20°) positioned randomly within a square aperture (15.18° width) and moving at 6.85°/s on a black screen (Fig. 5). Each dot had a limited lifetime of 667 ms (with a randomly chosen starting life), before being randomly replotted. On each trial, a proportion of the dots moved in the same direction (i.e., left or right), and the remaining dots moved in random directions. The stimuli and fixation point were displayed until response or after 5,000 ms had elapsed. Participants made their response (i.e., left or right) using the first fingers of each hand positioned on a button box.

As in Study 1, there were five blocks or ‘levels’. Level 1 started with an introduction to the task followed by four demonstration trials (100%, 100%, 75%, 50% coherence), which had no speed or accuracy emphasis. Then, the task instruction was introduced (“try to respond as [*accurately/quickly]* as possible”), and the researcher checked the participant’s understanding. Participants were then required to correctly respond to four consecutive trials within a maximum of 20 criterion trials with a coherence of 95%. Participants then completed eight practice trials of increasing difficulty (80%, 70%, 60%, 50%, 40%, 30%, 20%, 10%). Within this block, children were given feedback on the accuracy of their responses, as in Study 1, as well as feedback if they did not respond within 5,000 ms (“Timeout! Try to be quicker next time!”).

We then presented 38 trials in each of Levels 2 to 5, with 18 trials for each of two difficulty conditions (easy: 70% coherence; difficult: 25% coherence) and two catch trials (100% coherence), in random order. Difficulty levels were based on those used by Manning et al. (2022a; 30% and 75%), but slightly lower to minimise ceiling effects when asking children to emphasise accuracy. Half of the trials contained leftward coherent motion, and the other half had rightward coherent motion. Participants were encouraged to take a break at the end of each level. As in Study 1, trial-by-trial feedback was not given in Levels 2–5, but block-level feedback was given at the end of each block. Children were told: “the more you get right, the more points you will get”, or “the faster you are, the more points you will get”. In the accuracy-emphasis condition, points were awarded based on double the number of correct responses. In the speed-emphasis condition, each correct trial was given 1 point if it was faster than the mean response time in practice trials, and 2 points if it was faster than half the mean response time in practice trials. As before, children were always given a minimum of 10 points, to maintain motivation. The researcher reminded children of the speed–accuracy emphasis instruction before continuing the next block.

#### Model fitting

Unlike in Study 1, performance in catch trials were not used as an inclusion criteria at the blind modelling stage, as some individuals perceive motion in the opposite direction at 100% coherence (Manning et al., 2022c). However, two participants per group were removed at this stage because they did not reach 65% accuracy in the easy condition under accuracy-emphasis instructions. As in Study 1, we also excluded trials with response times <300 or >5,000 ms (.30% of trials).

We used the same modelling approach as in Study 1, with the same blinding process, priors, and chains. However, here *v.mean* became the average drift rate across difficulty levels *and* speed–accuracy instructions, and *a* and *ter* became the average boundary separation and non-decision time across speed–accuracy instructions. Following studies that have shown effects of speed–accuracy instructions on multiple parameters (Evans, 2021; Mulder et al., 2010, 2013; Zhang & Rowe, 2014), we incorporated parameters to model the difference in drift rate (*v.SAT*), boundary separation (*a.SAT*) and non-decision time parameters (*ter.SAT*) between the speed-emphasis and accuracy-emphasis conditions, with priors that matched those for *v.diff* in Study 1. Additionally, we included a model variant which modelled order effects (whether the speed-emphasis or accuracy-emphasis task was presented first):

- **Data level:**

$${y}_{pij} \sim diffusion\left({a}_{pj, } {z}_{p},{Ter}_{pj},{v}_{pij}, s\right)$$

- **Parameters:**

$$\begin{array}{lc}{a}_{p1}-{a}_{p2} \sim N\left({\mu}_{a.SAT} \pm {\delta}_{a.SAT} \pm {OE}_{a.SAT}, {\sigma}_{a.SAT}\right)\\ \frac{{a}_{p1}+ {a}_{p2}}{2}\sim N\left({\mu}_{a.mean} \pm {\delta}_{a.mean}, {\sigma}_{a.mean}\right)\\ {z}_{p}/{a}_{pj} \sim {TN}_{\mathrm{0,1}}\left({\mu}_{z} \pm {\delta}_{z}, {\sigma}_{z}\right)\\ {Ter}_{p1}-{Ter}_{p2} \sim N\left({\mu}_{Ter.SAT} \pm {\delta}_{Ter.SAT} \pm {OE}_{Ter.SAT}, {\sigma}_{Ter.SAT}\right)\\ \frac{{Ter}_{p1}+ {Ter}_{p2}}{2}\sim N\left({\mu}_{Ter.mean} \pm {\delta}_{Ter.mean}, {\sigma}_{Ter.mean}\right)\\ {(v}_{p11}+{v}_{p12})-{(v}_{p21}{ + v}_{p22}) \sim N\left({\mu}_{v.diff} \pm {\delta}_{v.diff}, {\sigma}_{v.diff}\right)\\ \left({v}_{p11}{+ v}_{p21}\right)-{(v}_{p12}+ {v}_{p22}) \sim N\left({\mu}_{v.SAT} \pm {\delta}_{v.SAT} \pm {OE}_{v.SAT}, {\sigma}_{v.SAT}\right)\\ \frac{{v}_{p11}+ {v}_{p12} + {v}_{p21} + {v}_{p22}}{4}\sim N\left({\mu}_{v.mean} \pm {\delta}_{v.mean}, {\sigma}_{v.mean}\right)\\ s=0.1\end{array}$$

- **Hyperparameters:**

$$\begin{array}{lc}{\mu}_{a.mean} \sim {N}_{+}\left(0.2, 0.2\right)\\{\mu}_{z} \sim {TN}_{\mathrm{0,1}}\left(0.5, 0.2\right)\\ {\mu}_{Ter.mean} \sim {N}_{+}\left(0.3, 0.3\right)\\ {\mu}_{v.diff},{\mu}_{v.SAT},{\mu}_{a.SAT},{\mu}_{Ter.SAT} \sim N\left(0, 0.1\right)\\ {\mu}_{v.mean} \sim N\left(0.3, 0.3\right)\\ {\sigma}_{a.SAT},{\sigma}_{a.mean},{\sigma}_{z},{\sigma}_{Ter.SAT},{\sigma}_{Ter.mean},{\sigma}_{v.SAT},{\sigma}_{v.diff},{\sigma}_{v.mean} \sim \Gamma \left(1, 1\right)\\ {\delta}_{a.SAT},{\delta}_{a.mean},{\delta}_{z},{\delta}_{Ter.SAT},{\delta}_{Ter.mean},{{\delta}_{v.SAT},\delta }_{v.diff},{\delta}_{v.mean} \sim N\left(0, 0.01\right)\\ {OE}_{a.SAT},{OE}_{Ter.SAT},{OE}_{v.SAT } \sim N\left(0, 0.1\right)\end{array}$$

where *j* now indexes speed–accuracy emphasis condition, and OE reflects the order effects parameter. As in Study 1, we ran further models which controlled for age and PIQ, modelled drift rate variability and modelled a contaminant process, to establish the robustness of our results. Our joint models were constructed to not only assess relationships between covariates and mean drift rate, boundary separation and non-decision time (as in Study 1), but also differences between these parameters across speed–accuracy emphasis instructions (i.e., *v.SAT*, *a.SAT*, *ter.SAT*). As in Study 1, we ensured that Gelman–Rubin diagnostic values for parameters underlying inferences (i.e. group-level parameters) were <1.1.

### Results and discussion

#### Accuracy and response time

**Fig. 6:**
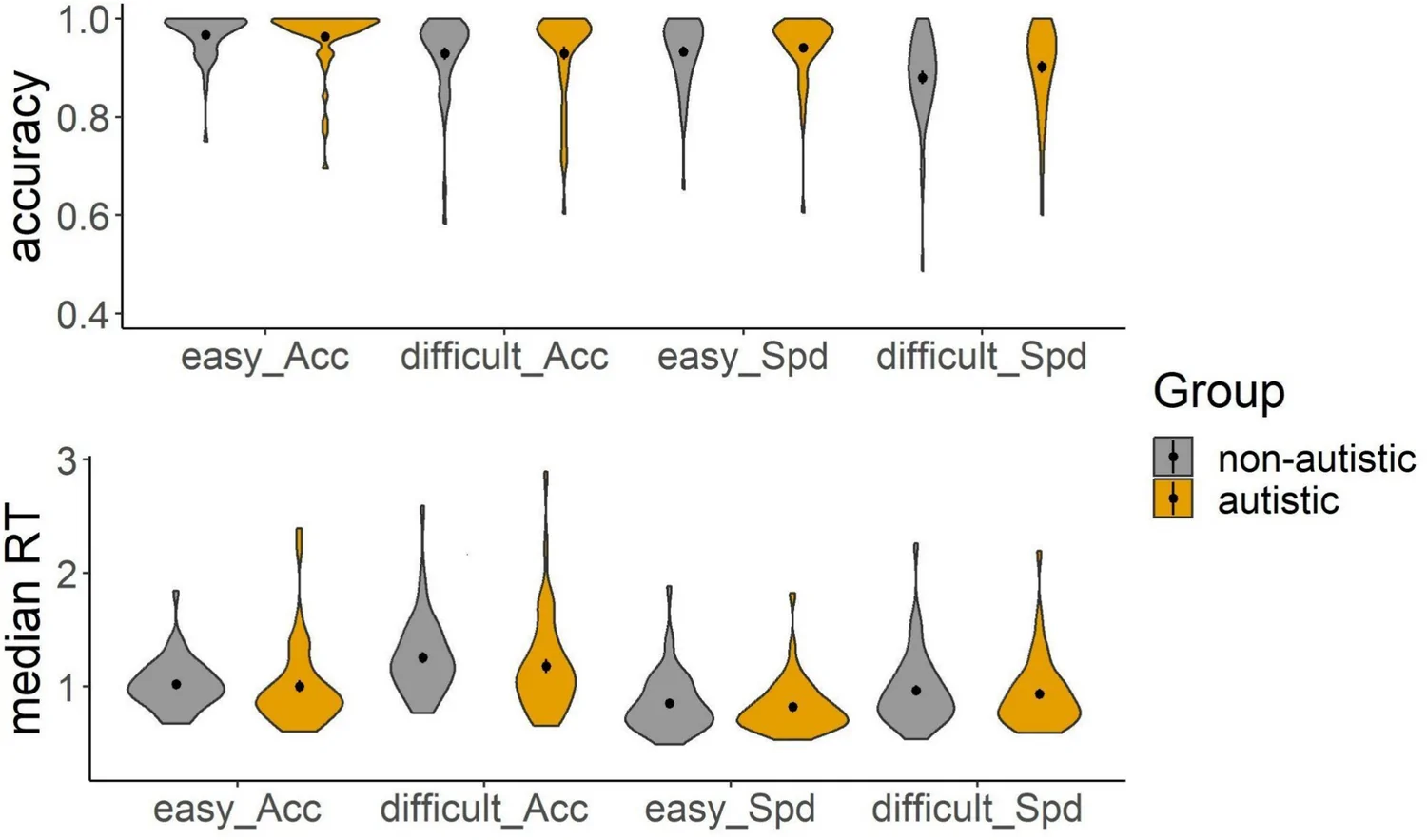
Violin plots of accuracy and median response time distributions for correct trials for each participant in the visual motion task with varying speed–accuracy emphasis instructions (Study 2). The grey and orange ‘violins’ reflect the kernel probability density of non-autistic and autistic children’s accuracy (upper panel) and median RT in seconds for correct trials (lower panel) for each difficulty level (easy: 70% coherence; difficult: 25% coherence) and instruction condition (Acc = accuracy emphasis; Spd = speed emphasis). Dots and vertical lines represent the group mean and ± 1 SEM. (Colour figure online)

The mean accuracy and mean of the median response times for autistic and non-autistic children are shown in Fig. 6. While not a preregistered analysis testing a hypothesis, a Bayesian factorial ANOVA conducted in JASP with default priors showed that lower accuracies were obtained in the difficult condition than the easy condition (BF_incl_ > 100), and when emphasising speed over accuracy (BF_incl_ > 100). Meanwhile there was no conclusive effect of group (BF_incl_ =.37) nor interactions with group and difficulty level (BF_incl_ =.36) or between group and speed–accuracy emphasis instruction (BF_incl_ =.61). Faster response times were obtained in the easy condition than the difficult condition (BF_incl_ > 100), and under speed emphasis than accuracy emphasis (BF_incl_ > 100), as expected. There was also an interaction between difficulty level and instruction (BF_incl_ > 100). Pairwise comparisons showed that there was a clear effect of difficulty level in both instruction conditions, although the difference in response time between easy and difficult conditions was more pronounced in the accuracy-emphasis condition (mean difference = .20 s) than the speed-emphasis condition (mean difference = .11 s). Meanwhile, there was no conclusive evidence for or against a group difference in response time (BF_incl_ = .48). There was conclusive evidence against an interaction between speed–accuracy emphasis instruction and group (BF_incl_ = .31) and inconclusive relationships for the other interaction terms (BF_incl_ = 1.17–1.27). Therefore, there were no clear differences between groups in either accuracy or response time. However, the main effects of instruction show that, overall, all children were able to modulate their performance according to the instructions.

#### Within-participants effects of speed–accuracy emphasis instructions on DDM parameters

**Fig. 7:**
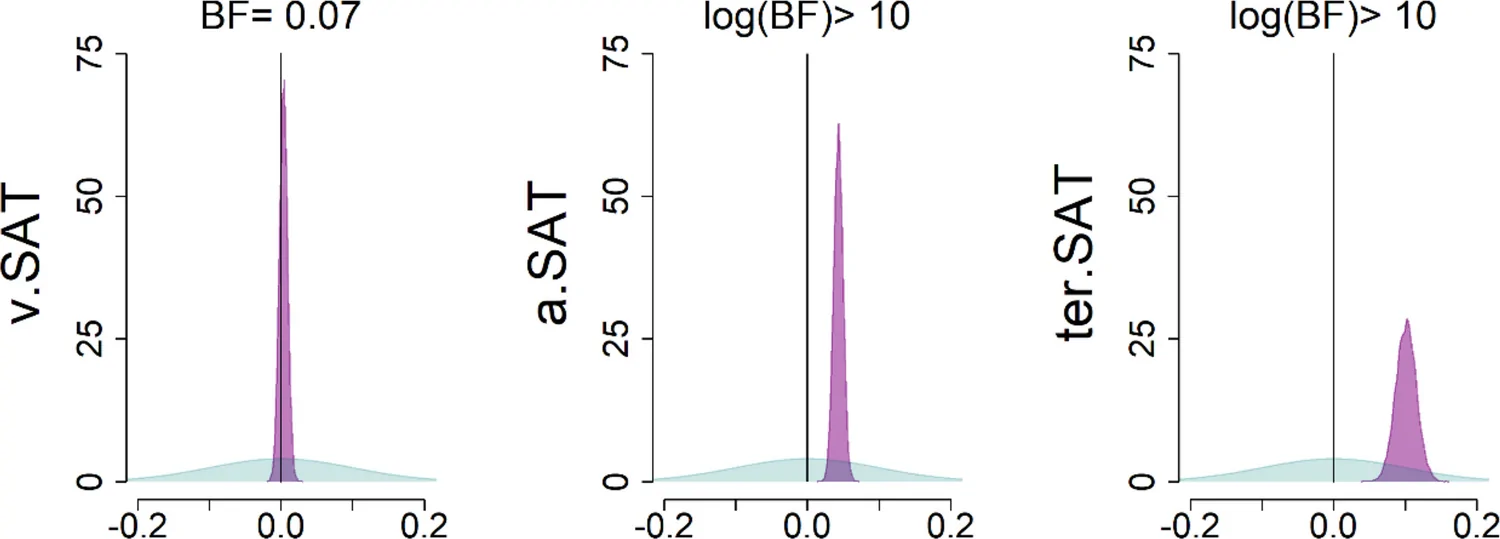
Prior (**blue**) and posterior (**purple**) density distributions for the main effect of speed–accuracy emphasis instructions on DDM parameters in Study 2. *Note. v.SAT* = mean difference in drift rate across speed–accuracy instructions; *a.SAT* = mean difference in boundary separation across speed–accuracy emphasis instructions; *ter.SAT* = mean difference in non-decision time across speed–accuracy emphasis instructions. BF = Savage–Dickey Bayes factors, where BF > 1 reflects relatively more support for the alternative hypothesis over the null hypothesis. (Colour figure online)

While we did not preregister hypotheses for the within-participants main effect of speed–accuracy emphasis instructions, there was strong evidence that both boundary separation and non-decision-time were modulated with instructions (BF > 100; Fig. 7). Under accuracy-emphasis instructions, children had longer non-decision times, and increased boundary separation. Meanwhile, there was conclusive evidence against effects of instructions on drift rate (BF = .07). The same pattern was also found in the autistic sample alone, with conclusive evidence for the effect of speed–accuracy instructions affecting non-decision times (BF > 100) and boundary separation (BF > 100), but not drift-rate (BF =.08).

#### Preregistered analyses: Group differences in DDM parameters

**Fig. 8:**
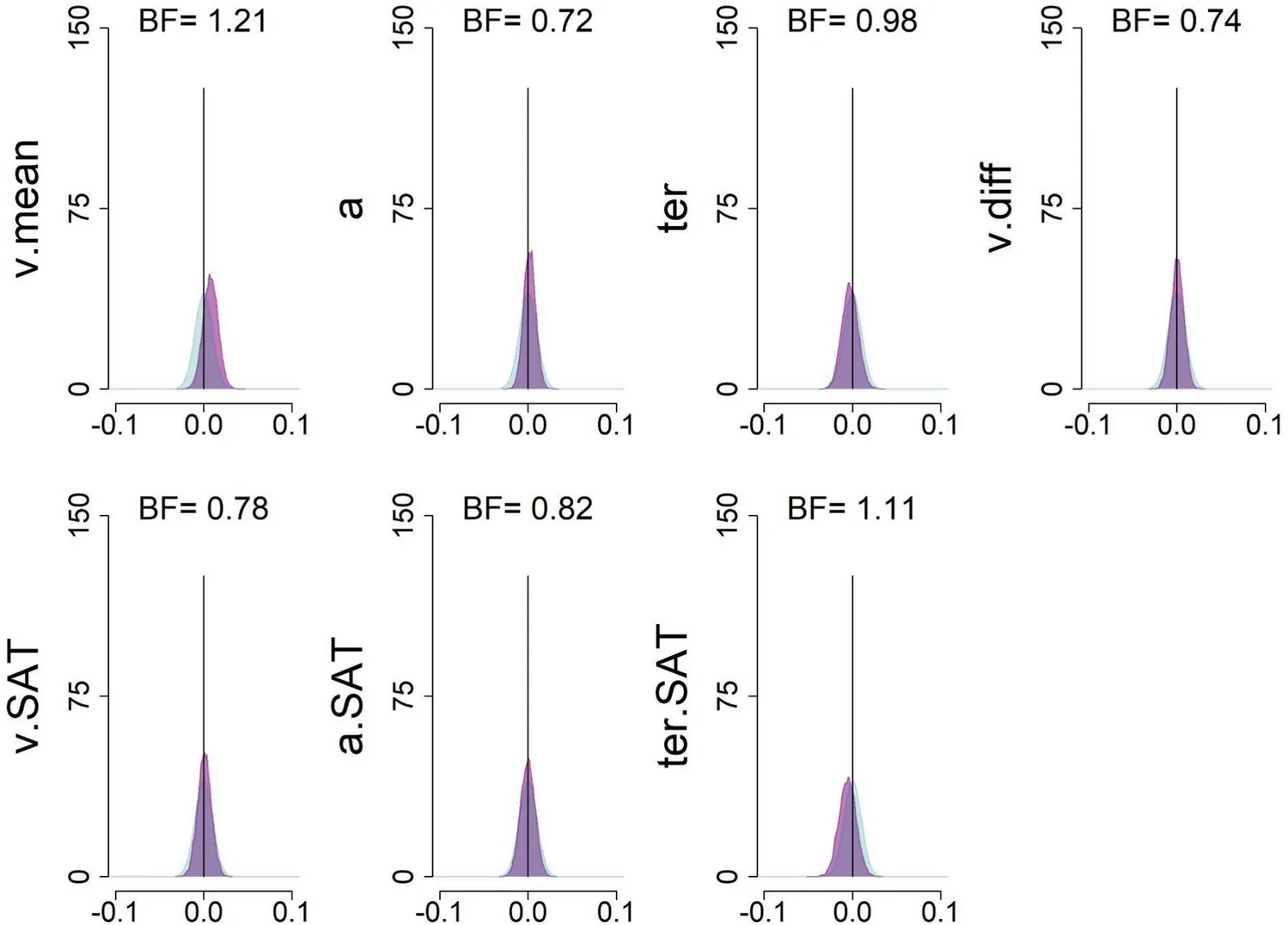
Prior (**blue**) and posterior (**purple**) density distributions for group differences in DDM parameters in Study 2. Posterior distributions shifted leftwards (negative) reflect lower values in the autistic group than the non-autistic group. The top row shows the distributions for the basic model without age and performance IQ (PIQ) partialled out. The bottom row shows the distributions for the model with age and PIQ partialled out. *Note. v.mean* = mean drift rate across difficulty levels and speed–accuracy instructions; *a* = mean boundary separation across speed–accuracy instructions; *ter* = mean non-decision time across speed–accuracy instructions; *v.diff* = mean difference in drift rate across difficulty levels; *v.SAT* = mean difference in drift rate across speed–accuracy instructions; *a.SAT* = mean difference in boundary separation across speed–accuracy emphasis instructions; *ter.SAT* = mean difference in non-decision time across speed–accuracy emphasis instructions. BF = Savage–Dickey Bayes factors, where BF > 1 reflects relatively more support for the alternative hypothesis over the null hypothesis. (Colour figure online)

In the basic model, there was no conclusive evidence for or against group differences (Fig. 8). There was relatively more evidence for the null hypothesis for boundary separation (BF =.72), non-decision time (BF =.98), difference in drift rate across difficulty levels (BF =.74), difference in drift rate across speed–accuracy emphasis instructions (BF =.78), and difference in boundary separation across speed–accuracy emphasis instructions (BF =.82). There was relatively more evidence for the alternative hypothesis for group differences in mean drift rate (BF = 1.21), and differences in non-decision time across speed–accuracy emphasis instructions (BF = 1.11), but this constituted very weak, inconclusive evidence. The same pattern of results was found for models which partialled out age and PIQ, modelled an order effect (whether speed emphasis or accuracy emphasis was presented first), drift rate variability, and a contaminant process (Supplementary Table 6).

#### Exploratory analyses: Group differences in DDM parameters with non-hierarchical modelling

Following these inconclusive results, we also ran individual non-hierarchical DDM fits to investigate how this might affect our inferences, as in Study 1. We conducted a Bayesian ANCOVA on posterior mean drift rate estimates for each participant, with within-participants effects of difficulty level (easy, difficult), speed–accuracy instructions condition (speed emphasis, accuracy emphasis), a between-participants effect of group, and PIQ as a covariate (as in Study 1). The best model was one with group, difficulty level and PIQ included. The inclusion Bayes factor for group was 1.26, showing inconclusive evidence for the effect of group, with the autistic children having slightly higher drift rates than the non-autistic children. We then ran Bayesian ANCOVAs with group and speed–accuracy instructions and PIQ as a covariate for boundary separation and non-decision time. For boundary separation, the best model was one that included just the main effect of speed–accuracy instructions, with conclusive evidence against the inclusion of group (BF_incl_ = .25) and the interaction between group and instructions (BF_incl_ = .22). For non-decision time, the best model also only included the main effect of speed–accuracy instruction, with no conclusive evidence for group differences (BF_incl_ = .42) or interaction between speed–accuracy instruction and group (BF_incl_ = .87). Therefore, many group differences remain inconclusive with this different analytical approach, apart from the conclusive evidence against group differences in boundary separation. Note that we found conclusive evidence that speed–accuracy instruction affected boundary separation and non-decision time, but not drift rate, as in our hierarchical analyses (Fig. 7).

#### Preregistered analyses: Relationships between DDM parameters and ADHD traits

We hypothesised that the difference in boundary separation across speed–accuracy instructions would be negatively related to hyperactivity/impulsivity. However, across the whole sample, using our basic model, we found inconclusive evidence for or against this relationship (posterior mean correlation = −.16, 95% CI [−.36, .06], BF = .38), with relatively more evidence in support of the null hypothesis. A similar result was obtained when modelling drift rate variability and a contaminant process, although the evidence was conclusively in support of the null hypothesis when partialling out age and PIQ (Supplementary Table 7).

Across the whole sample, we found conclusive evidence against the hypothesised relationship between hyperactivity/impulsivity and drift rate (posterior mean correlation = −.11, 95% CI [−.30, .09], BF = .23). Conclusive evidence was also found against relationships between hyperactivity/impulsivity and mean boundary separation (posterior mean correlation =.03, 95% CI [−.17, .24], BF = .15), and hyperactivity/impulsivity and the difference in drift rate across speed–accuracy instructions (posterior mean correlation = −.04, 95% CI [−.25, .17], BF = .15). These results were the same across all model variants (Supplementary Table 7). There was also a conclusive negative relationship between hyperactivity/impulsivity and mean non-decision time (posterior mean correlation = −.25, 95% CI [−.43, −.06], BF = 3.21) in the basic model, which was particularly supported when partialling out age and PIQ (BF = 24.68), but inconclusive when modelling a contaminant process (BF = .45; Supplementary Table 7). The relationship between hyperactivity/impulsivity and difference in non-decision time across speed–accuracy instructions (posterior mean correlation = −.15, 95% CI [−.34, .06], BF = .37) was inconclusive but with more evidence for the null hypothesis (in all model variants, Supplementary Table 7).

We also did not find support for the hypothesised relationship between hyperactivity/impulsivity and difference in boundary separation across speed–accuracy instructions when looking at each group separately (non-autistic group: BF = .39; autistic group: BF = .28; Supplementary Table 8). There was conclusive evidence against the hypothesis of a negative relationship between drift rate and hyperactivity/impulsivity in the autistic group (posterior mean correlation = −.14, 95% CI [.37, .10], BF = .30). No conclusive evidence was found for or against any relationships between DDM parameters and hyperactivity/impulsivity in each group separately (Supplementary Table 8).

Across the whole sample, there was conclusive evidence against relationships between inattention and drift rate (posterior mean correlation = −.09, 95% CI [−.28, .12], BF = .18), boundary separation (posterior mean correlation = −.04, 95% CI [−.24, .17], BF =.15), difference in drift rate across speed–accuracy instructions (posterior mean correlation = −.05, 95% CI [−.26, .16], BF = .16), difference in boundary separation across speed–accuracy conditions (posterior mean correlation = −.07, 95% CI [−.28, .14], BF = .17) and difference in non-decision time across speed–accuracy conditions (posterior mean correlation = −.08, 95% CI [−.28, .13], BF = .18). The same pattern was found across all model variants (Supplementary Table 7), apart from the relationship between inattention and difference in boundary separation across speed–accuracy conditions, which was inconclusive when modelling a contaminant process (BF =.37). The relationship between inattention and non-decision time was inconclusive across the whole sample, with relatively more evidence for the alternative hypothesis (posterior mean correlation = −.23, 95% CI [−.41, −.03], BF = 1.62). This relationship was conclusive when partialling out age and PIQ (BF = 3.10), but not evident in the model which modelled a contaminant process (where there was more evidence for the null hypothesis, BF = .24). When investigating correlations in each group separately, there was no conclusive evidence for any relationships between inattention and DDM parameters (BF ≤ .77, Supplementary Table 8).

#### Exploratory analyses: Relationships between DDM parameters and other individual differences measures

There was conclusive evidence against relationships between DCDQ and DDM parameters using the basic model (Supplementary Table 9). This pattern of results was mostly consistent when controlling for age and PIQ, although the relationship between DCDQ and difference in non-decision time across speed–accuracy instructions became inconclusive, but with more evidence for the null hypothesis (BF = .36). There was also conclusive evidence against relationships between sensory overresponsivity and DDM parameters, both with and without controlling for age and PIQ (Supplementary Tables 9 and 10). There was no conclusive evidence for relationships between sensory underresponsivity and DDM parameters, although there was relatively more evidence for the alternative hypothesis between sensory underresponsivity and non-decision time when controlling age and PIQ (posterior mean correlation = −.23, 95% CI [−.41, −.04], BF = 1.81). All other Bayes factors for relationships between sensory underresponsivity and DDM parameters were under 1.

There was a conclusive, negative relationship between TOWRE sight-word efficiency and boundary separation (posterior mean correlation = −.27, 95% CI [−.45, −.07], BF = 4.36), including when controlling for age and PIQ (posterior mean correlation = −.27, 95% CI [−.45, .08], BF = 5.49). The relationship between TOWRE sight-word efficiency and non-decision time was inconclusive in the basic model, but with relatively more evidence for the alternative hypothesis (posterior mean correlation = −.24, 95% CI [−.42, −.04], BF = 1.98). This relationship became conclusive when controlling for age and PIQ (posterior mean correlation = −.27, 95% CI [−.45, −.07], BF = 5.15). There was no evidence for any other relationships between sight-word efficiency and DDM parameters (BF < .40). Finally, we found no conclusive evidence for relationships with TOWRE phonemic decoding efficiency in the basic model, both with and without controlling for age and PIQ (all BF ≤ .83).

### Discussion of Study 2 results

To summarise our results, we found that autistic and non-autistic children can update their task performance in line with instructions to emphasise either speed or accuracy. Specifically, they increased their boundary separation and non-decision time when asked to emphasise accuracy. These results align with previous studies reporting effects on boundary separation and non-decision time (Mulder et al., 2010, 2013; Voss et al., 2004), as well as a reanalysis of previous studies showing that instructions emphasising either speed or accuracy typically only effects boundary separation and non-decision time when analysed using the DDM (Evans, 2021). We were most interested in group differences in this ability, but the data were not sufficiently diagnostic to distinguish between the presence or absence of group differences: all group differences for parameters modelling the difference across speed-emphasis and accuracy-emphasis instructions were inconclusive in our hierarchical models. Our results show, however, for the first time, that autistic children can update their decision-making strategies in perceptual decision-making tasks when asked to emphasise either speed or accuracy. These results add to other studies suggesting that cognitive flexibility is not uniformly reduced in autistic participants (Leung & Zakzanis, 2014; Manning et al., 2017). Yet, these results stand in contrast to a previous report of reduced ability to modulate boundary separation in response to task instructions in children with ADHD (Mulder et al., 2010). This difference could imply a difference between children with ADHD and autistic children, although this would need testing explicitly in large samples. We also considered that our inconclusive results could be due to our hierarchical Bayesian modelling approach, as Mulder et al. (2010) used a two-step approach where they fit the DDM to individual participants and then conducted an ANOVA on the resulting estimates. With non-hierarchical (participant-level) modelling, and inferential statistics conducted on point estimates, we found conclusive evidence against an interaction between group and speed–accuracy instructions on boundary separation. While this does not affect our conclusions, it suggests that this inference approach does lead to more firm conclusions in some cases, which we will reflect on in the General Discussion.

We also hypothesised that autistic children would show increased boundary separation. However, as in Study 1, we found no conclusive evidence for or against differences in boundary separation in our hierarchical models, with relatively more evidence for the null hypothesis (BF =.71 in Study 1, and BF =.72 in Study 2) of no group differences. Evidence for group differences in drift-rate and non-decision time was also inconclusive. We had considered the possibility that the results would be inconclusive, as they were in Manning, Hassall, Hunt, Norcia, Wagenmakers, Evans et al. (2022a), with a similar sample and task (although Manning et al. gave instructions to balance both speed and accuracy). Our non-hierarchical models and inferential statistics on point estimates varied from showing conclusive evidence against group differences (for boundary separation), to showing inconclusive evidence for both drift rate and non-decision time.

We found no conclusive evidence for or against the hypothesised relationship between difference in boundary separation across task instructions and hyperactivity/impulsivity ADHD traits, in either the whole sample, or in each group separately, with relatively more evidence for the null. Similarly, we found no conclusive evidence that drift rates were negatively related to hyperactivity/impulsivity in the whole sample, and in the autism group alone, there was conclusive evidence against this relationship (in contrast to Manning, Hassall, Hunt, Norcia, Wagenmakers, Evans et al., 2022a). We cannot be sure why we did not replicate the relationship for Manning et al., although we note that we used a slightly different ADHD measure here. Future research could therefore compare the two ADHD measures. The only relationship between DDM parameters and hyperactivity/impulsivity which showed conclusive evidence was that between non-decision time and hyperactivity/impulsivity in the basic model. Interestingly, this was the same relationship recently identified in a study of autistic and non-autistic adult’s numerosity discrimination (Yusuf et al., 2025). However, this relationship became inconclusive when modelling a contaminant process. This attenuation is consistent with prior work showing that contaminant-modelling can reallocate extreme RTs away from the decision process (Ratcliff & Tuerlinckx, 2002), reducing the variance attributed to non-decision time.

Finally, our exploratory analyses suggested a conclusive negative relationship between TOWRE sight-word efficiency and boundary separation, and when controlling for age and PIQ, a conclusive negative relationship between TOWRE sight-word-efficiency and non-decision time. Similarly, O’Brien and Yeatman (2021) reported negative relationships between reading ability and boundary separation, and reading ability and non-decision time in children during a motion coherence task, where reading ability was a composite including TOWRE and Woodcock Johnson IV scores. However, we did not find conclusive evidence for other relationships reported by O’Brien and Yeatman (2021), including between drift rate and reading ability (see also Manning et al., 2022b who showed selective differences in drift rate between children with and without dyslexia). The fact that we found relationships in Study 2 but not Study 1 could be due to specific relationships between reading and motion processing (in line with the magnocellular theory (Stein, 2019; Stein & Walsh, 1997). However, it is worth noting that in Study 1, the relationship between TOWRE SWE and boundary separation was inconclusive, but with more evidence for the alternative hypothesis, and a very similar posterior mean correlation. Therefore, it may be that there is a relationship between reading ability and DDM parameters in an orientation task, although potentially weaker than that for the motion task: a possibility that needs to be tested in future confirmatory work.

## General discussion

Across two studies with different perceptual decision-making tasks, we find little evidence for or against group differences in autistic and non-autistic children’s decision-making parameters. One of the most commonly reported differences between autistic and non-autistic participants in previous DDM studies is that of increased boundary separation in autism. Yet our results add to a growing body of studies which have not reported such a difference, including a study of autistic children’s motion processing (Manning, Hassall, Hunt, Norcia, Wagenmakers, Evans et al., 2022a), a study of autistic adults’ numerosity discrimination (Yusuf et al., 2025), and a study of autistic adolescents’ face processing, which instead reported narrower boundary separation (Powell et al., 2019). In the current paper, we aimed to better understand why this could be the case. First, we considered whether it could be due to the choice of task, with previous reports of increased boundary separation in orientation tasks. However, here we found no conclusive evidence of increased boundary separation both in an orientation task, similar to that of Pirrone et al. (2020), and a motion coherence task. It therefore did not appear to matter whether the task was more reliant on local processing, and form-based (as in the orientation task), or more reliant on global processing, and motion-based (as in the motion coherence task), despite theories of altered local-global processing in autism (Happé & Frith, 2006; Mottron et al., 2006) and dorsal-stream vulnerability (Braddick et al., 2003).

Second, we considered whether it was due to differences in task instructions, with the idea that autistic participants may show a general tendency towards more accurate responding, but can balance speed and accuracy when explicitly instructed to. Study 2 shows that autistic and non-autistic children can indeed update their decision-making strategies when asked to either emphasise speed or accuracy, showing the importance of task instructions. However, autistic children also did not show increased boundary separation in Study 1, when given *no* explicit task instructions to be fast or accurate. Therefore, we do not think that task instructions can fully explain the different results across previous studies. However, we note that many previous studies have not clearly reported the instructions given to participants in terms of speed–accuracy emphasis (e.g., Iuculano et al., 2020; Karalunas et al., 2018; Pirrone et al., 2017, 2020). Our results show that this is an important omission, given the influence of task instructions on children’s DDM parameters. We therefore encourage future studies to explicitly report their task instructions and how feedback is given, and ideally to share experimental code.

Finally, we considered whether the modelling approach might be important. Most studies showing group differences have taken a two-step approach, first fitting DDMs to individual participant data, and then making inferences based on the participant-level parameter estimates. Our hierarchical modelling approach instead modelled both participant-level and group-level parameters, and we extracted Bayes factors based on the full posterior distributions for group-level difference parameters. The inferences made therefore preserve uncertainty at both the participant and group level. To investigate whether this could be contributing to differences between studies, we also ran non-hierarchical DDMs and conducted statistical models on the resulting parameter estimates, as in Pirrone et al. (2020). Here, certain effects became more conclusive, suggesting that this could contribute to the more conclusive results previously reported. However, even with this different approach, we did not find any conclusive evidence of increased response caution. Therefore, we still have not fully solved the different pattern of results. However, this two-step approach could exaggerate evidence, either for the null or alternative hypothesis (Boehm et al., 2018). Future work could further investigate the impact of modelling approach and task differences. It would be informative to remodel the data from previous studies with our single-step hierarchical approach, to see whether this shows increased response caution.

It seems that while evidence for group differences in boundary separation in perceptual decision-making tasks is not always found, there are other examples suggesting more cautious decisions by autistic people in other types of decision-making tasks (e.g., Brosnan et al., 2014; Vella et al., 2018; Wilson & Bishop, 2022). After collecting the results reported here, we held a consultation with five autistic community members, who generally felt that they did face decision-making difficulties in daily life (see also Luke et al., 2012), and who suggested reasons why we may not have found differences in our perceptual decision-making studies. This consultation suggested that increased caution might depend on the number of choice options, the importance of the decision, and other contextual factors. Similarly, a narrative review proposed that decision-making is similar across autistic and non-autistic people for simple perceptual tasks, but with group differences emerging for more complex metacognitive and value-based tasks (van der Plas et al., 2023). Future work could therefore aim to better understand the conditions under which increased response caution in autistic individuals might emerge.

Nonetheless, our results contribute to our understanding of decision-making in autism, by showing that autistic children can flexibly update their decision-making strategies following speed–accuracy instructions. Executive functioning theories (Hughes et al., 1994; Ozonoff et al., 1994) suggest that autism is characterised by reduced cognitive flexibility and difficulty with strategy-switching. While our results do not conclusively show the absence or presence of group differences, they do highlight that autistic children are able to modulate their decision-making strategies, suggesting that cognitive flexibility is not uniformly reduced in autism. The result is also relevant to proposals of inflexible decision rules in autism (Hadad & Yashar, 2022) and high and inflexible precision of prediction errors (Van de Cruys et al., 2014). Modulation of the speed–accuracy trade-off has been linked to activity in the striatum and presupplementary motor area (Forstmann et al., 2008, 2010) and alterations in striatal circuitry have been implicated in autism (Li & Pozzo-Miller, 2020). It would therefore be informative to study the neural correlates of the speed–accuracy trade-off in autistic and non-autistic children, to determine whether they are using the same or different neural pathways to modulate their decision-making strategies.

Another aim of the study was to better understand the considerable variability in decision-making parameters reported in previous developmental studies (Manning et al., 2021; Manning, Hassall, Hunt, Norcia, Wagenmakers, Evans et al., 2022a; Manning, Hassall, Hunt, Norcia, Wagenmakers, Snowling et al., 2022b). There was some evidence for relationships between DDM parameters and standardised measures of individual differences, including TOWRE sight-word reading efficiency. However, relationships were not always consistent across all model variants tested, and were not conclusive, even for relationships that have previously been reported in children (e.g., Manning, Hassall, Hunt, Norcia, Wagenmakers, Evans et al., 2022a; O’Brien & Yeatman, 2021). It is possible that covariates have interacting effects on DDM parameters, which was beyond the scope of the current project. However, developing hierarchical modelling approaches which investigate relationships between different covariates would be helpful to better understand individual variability across children.

We also noted that subtly different patterns of results were apparent with and without controlling for age and performance IQ. While controlling for age is standard practice, and perhaps not contentious, what it means to control for PIQ when assessing group or individual-level differences in parameters needs careful thought (Dennis et al., 2009). Certain DDM parameters like drift rate are known to be linked to PIQ (Lerche et al., 2020), and drift rate and PIQ may be related to our group or individual-level differences in important ways. Controlling for PIQ may therefore take out some of the variance in the effects that we are interested in. Moreover, the fact that we show subtly different patterns of results with and without controlling for age and PIQ suggests that the decision to control for covariates or not could introduce differences in results across studies. We therefore suggest that future studies report findings both with and without PIQ as a covariate. Future work may also benefit from investigating age and cognitive abilities as concepts of interest in autism, rather than just as covariates, as this could help us understand how developmental trajectories of perceptual decision-making are shaped by neurodevelopmental processes. For example, risky decision-making develops differently between 11 and 14 years of age in autistic and non-autistic young people (Hosozawa et al., 2021).

Overall, given the many inconclusive results reported in this study, larger samples may be needed to reach firm conclusions about group differences between autistic and non-autistic participants, and relationships with measures of individual differences. This is, of course, difficult to achieve practically, and may necessitate collaborative data collection efforts across sites. Future research could also benefit from using ‘informed priors’ based on previous work assessing group differences. Given the many different possible ways of fitting DDMs to the data, we feel that the preregistration and blind-modelling steps we took are particularly important for improving transparency and reducing publication bias. We end, therefore, with a recommendation for future work using DDMs to include large samples, with preregistered analysis approaches, and clear reporting of task instructions, when investigating group differences in autism or individual differences across development.

## Supplementary Information

Below is the link to the electronic supplementary material.

Supplementary file1 (DOCX 38 KB)

## Data Availability

De-identified data are available in a safeguarded repository on the UK Data Service (https://doi.org/10.5255/UKDA-SN-858223).

## Code availability

Experimental code, analysis scripts and output files for Study 1 (https://osf.io/vfqwx/) and Study 2 (https://osf.io/2gyc4/).
