# Supplementary material for: Autistic and non-autistic children’s perceptual decision-making in visual orientation and motion tasks and the effect of task instructions
Source: Atten Percept Psychophys. 2026 Sep 6;88(7):188. doi: 10.3758/s13414-026-03330-8 (PMC13547164; doi:10.3758/s13414-026-03330-8)
Supplement: Supplementary file 1 [file 13414_2026_3330_MOESM1_ESM.docx]

**Supplementary Materials**

**Supplementary Table 1.** Gelman-Rubin statistics and Bayes factors for group differences in diffusion model parameters across different model variants for the visual orientation task (Study 1).

| **Model variant** | **Partialling out age and PIQ** | **Gelman-Rubin statistic (range)** | ***Bayes factor for group difference*** | | | |
| --- | --- | --- | --- | --- | --- | --- |
|  |  |  | ***v*** | ***a*** | ***ter*** | ***v.diff*** |
| Basic | No | 1.001 - 1.004 | 0.69 | 0.71 | 1.00 | 1.48 |
| Basic | Yes | 1.001 - 1.004 | 0.64 | 0.71 | 1.02 | 3.23 |
| Drift rate variability | No | 1.000 - 1.004 | 0.74 | 0.74 | 1.03 | 1.39 |
| Contaminant process | No | 1.000 - 1.004 | 0.74 | 0.73 | 0.97 | 1.71 |
| Drift rate variability | Yes | 1.001 - 1.004 | 0.64 | 0.71 | 0.98 | 2.85 |
| Contaminant process | Yes | 1.002 - 1.003 | 0.70 | 0.73 | 1.04 | 2.60 |

*Note. v* = drift rate, *a* = boundary separation, *ter* = non-decision time, *v.diff* = difference in drift rate between easy and difficult conditions. The range of Gelman-Rubin statistics is provided for the parameters of interest (i.e., group-level parameters).

**Supplementary Table 2.**

*Gelman-Rubin statistics and correlations between hyperactivity/impulsivity and inattention scores and diffusion model parameters for additional models in Study 1.*

| Model | Gelman-Rubin statistic  (range) | *v* | | *a* | | *ter* | |
| --- | --- | --- | --- | --- | --- | --- | --- |
|  |  | Correlation* | BF | Correlation | BF | Correlation | BF |
| *Hyperactivity/impulsivity* | | | | | | | |
| Between-trial variability in drift rate (*sv*) | 1.000 - 1.001 | -.08 [-.27, .13] | .17 | .00 [-.21, .20] | .14 | -.13 [-.32, .07] | .28 |
| Contaminant process | 1.000 - 1.001 | -.06 [-.26, .14] | .16 | -.06 [-.26, .15] | .15 | -.01 [-.21, .20] | .13 |
| Partial (age and PIQ) | 1.000 - 1.001 | .00 [-.21, .20] | .14 | -.10 [-.30, .10] | .22 | -.16 [-.35, .05] | .43 |
| *Inattention* | | | | | | | |
| Between-trial variability in drift rate (*sv*) | 1.000 - 1.001 | -.05 [-.25, .15] | .15 | -.05 [-.25, .15] | .15 | -.11 [-.30, .09] | .22 |
| Contaminant process | 1.001 - 1.001 | -.04 [-.24, .16] | .15 | -.08 [-.28, .14] | .18 | .01 [-.19, .22] | .14 |
| Partial (age and PIQ) | 1.000 - 1.001 | -.06 [-.27, .14] | .16 | -.19 [-.38, .02] | .70 | -.21 [-.40, -.01] | 1.06 |

*Note. v* = drift rate, a = boundary separation, *ter* = non-decision time. BF = Bayes factor. *The posterior mean correlation and 95% credible intervals are presented for each correlation between ADHD subscale (inattention, hyperactivity/impulsivity) score and diffusion model parameter (*v*, *a*, *ter*). The range of Gelman-Rubin statistics is provided for the parameters of interest (i.e., covariates).

**Supplementary Table 3.**

*Gelman-Rubin statistics and correlations between total hyperactivity/impulsivity and inattention scores and diffusion model parameters in each group for Study 1.*

| Model | Gelman-Rubin statistic  (range) | *v* | | *a* | | *ter* | |
| --- | --- | --- | --- | --- | --- | --- | --- |
|  |  | Correlation* | BF | Correlation | BF | Correlation | BF |
| *Hyperactivity/impulsivity* | | | | | | | |
| Non-autistic group | 1.001 - 1.004 | -.23 [-.51, .12] | .60 | -.05 [-.34, .25] | .21 | -.07 [-.33, .22] | .20 |
| Autistic group |  | -.06 [-.31, .19] | .19 | -.04 [-.31, .23] | .20 | -.26 [-.50, .02] | .94 |
| *Inattention* | | | | | | | |
| Non-autistic group | 1.001 - 1.003 | -.05 [-.40, .33] | .27 | -.21 [-.48, .11] | .50 | -.10 [-.38, .20] | .24 |
| Autistic group |  | -.10 [-.33, .14] | .23 | -.13 [-.38, .14] | .28 | -.26 [-.49, .00] | 1.23 |

*Note. v* = drift rate, a = boundary separation, ter = non-decision time. BF = Bayes factor. *The posterior mean correlation and 95% credible intervals are presented for each correlation between ADHD subscale (inattention, hyperactivity/impulsivity) score and diffusion model parameter (*v*, *a*, *ter*). The range of Gelman-Rubin statistics is provided for the parameters of interest (i.e., covariates).

**Supplementary Table 4.**

*Gelman-Rubin statistics and correlations between diffusion model parameters and DCDQ, sensory processing, and TOWRE scores across the whole sample in basic joint models for Study 1.*

| Covariate | Gelman-Rubin statistic  (range) | *v* | | *a* | | *ter* | |
| --- | --- | --- | --- | --- | --- | --- | --- |
|  |  | Correlation* | BF | Correlation | BF | Correlation | BF |
| DCDQ | 1.000 - 1.001 | .04 [-.16, .24] | .14 | .06 [-.14, .27] | .15 | .00 [-.21, .20] | .14 |
| Sensory OR | 1.000 - 1.001 | .06 [-.15, .25] | .15 | .11 [-.10, .31] | .25 | -.07 [-.26, .13] | .16 |
| Sensory UR | 1.001 - 1.001 | .05 [-.15, .25] | .15 | -.21 [-.40, -.01] | 1.06 | -.07 [-.26, .14] | .16 |
| TOWRE SWE | 1.000 - 1.001 | .05 [-.15, .25] | .15 | -.24 [-.43, -.03] | 1.70 | -.16 [-.35, .04] | .47 |
| TOWRE PDE | 1.000 - 1.001 | .00 [-.20, .20] | .14 | -.13 [-.32, .08] | .28 | -.05 [-.25, .15] | .15 |

**Supplementary Table 5.**

*Gelman-Rubin statistics and correlations between diffusion model parameters and DCDQ, sensory processing, and TOWRE scores across the whole sample while controlling for age and performance IQ in Study 1.*

| Covariate | Gelman-Rubin statistic  (range) | *v* | | *a* | | *ter* | |
| --- | --- | --- | --- | --- | --- | --- | --- |
|  |  | Correlation* | BF | Correlation | BF | Correlation | BF |
| DCDQ | 1.000 - 1.001 | .10 [-.11, .30] | .21 | .15 [-.05, .34] | .36 | .23 [.03, .41] | 1.58 |
| Sensory OR | 1.000 - 1.001 | .01 [-.19, .22] | .14 | .02 [-.18, .22] | .13 | -.16 [-.35, .04] | .47 |
| Sensory UR | 1.000 - 1.001 | .25 [.04, .43] | 1.98 | -.06 [-.26, .14] | .16 | -.28 [-.45, -.08] | 6.15 |
| TOWRE SWE | 1.000 - 1.001 | .16 [-.05, .35] | .41 | -.18 [-.36, .03] | .55 | -.17 [-.36, .03] | .56 |
| TOWRE PDE | 1.001 - 1.001 | .14 [-.07, .33] | .32 | -.12 [-.32, .09] | .27 | -.04 [-.24, .17] | .14 |

**Supplementary Table 6.**

Gelman-Rubin statistics and Bayes factors for group differences in diffusion model parameters across additional model variants for the visual motion task with varying speed-accuracy emphasis instructions (Study 2).

| **Model variant** | **Gelman-Rubin statistic (range)** | ***Bayes factor for group difference*** | | | | | | |
| --- | --- | --- | --- | --- | --- | --- | --- | --- |
|  |  | ***v*** | ***a*** | ***ter*** | ***v.diff*** | ***v.SAT*** | ***a.SAT*** | ***ter.SAT*** |
| Partial (age & PIQ) | 1.001 - 1.004 | 1.36 | .71 | .91 | .68 | .78 | .80 | 1.16 |
| Order effect | 1.001 - 1.004 | 1.32 | .70 | .95 | .76 | .80 | .84 | 1.10 |
| Drift rate variability | 1.000 - 1.004 | 1.27 | .67 | .97 | .76 | .79 | .82 | 1.07 |
| Contaminant process | 1.001 - 1.004 | 1.34 | .82 | .96 | .80 | .88 | .92 | 1.21 |

*Note. v* = drift rate, *a* = boundary separation, *ter* = non-decision time, *v.diff* = difference in drift rate between easy and difficult conditions, *v.SAT* = difference in drift rate across speed-accuracy emphasis instruction conditions, *a.SAT* = difference in boundary separation across speed-accuracy emphasis instruction conditions, *ter.SAT* = difference in non-decision time across speed-accuracy emphasis instruction conditions, PIQ = performance IQ. The range of Gelman-Rubin statistics is provided for the parameters of interest (i.e., group-level parameters).

**Supplementary Table 7.**

*Correlations between hyperactivity/impulsivity and inattention scores and diffusion model parameters for additional models in Study 2.*

| Model | *v* | | *a* | | *ter* | | *v.SAT* | | *a.SAT* | | *ter.SAT* | |
| --- | --- | --- | --- | --- | --- | --- | --- | --- | --- | --- | --- | --- |
|  | Correlation* | BF | Correlation | BF | Correlation | BF | Correlation* | BF | Correlation | BF | Correlation | BF |
| *Hyperactivity/impulsivity* | | | | | | | | | | | | |
| Between-trial variability in drift rate (*sv*) | -.11 [-.30, .09] | .24 | .03 [-.17, .24] | .14 | -.26 [-.43, -.06] | 2.90 | -.04 [-.25, .18] | .15 | -.16 [-.36, .06] | .37 | -.15 [-.34, .06] | .37 |
| Contaminant process | -.12 [-.32, .08] | .27 | -.03 [-.23, .18] | .14 | -.17 [-.36, .04] | .45 | -.03 [-.24, .20] | .15 | -.18 [-.38, .04] | .50 | -.18 [-.38, .03] | .56 |
| Partial (age and PIQ) | .00 [-.20, .21] | .13 | .03 [-.17, .23] | .14 | -.32 [-.49, -.13] | 24.68 | -.07 [-.28, .14] | .17 | -.01 [-.22, .20] | .14 | -.14 [-.33, .06] | .33 |
| *Inattention* | | | | | | | | | | | | |
| Between-trial variability in drift rate (*sv*) | -.09 [-.28, .12] | .19 | -.04 [-.24, .17] | .15 | -.23 [-.41, -.03] | 1.48 | -.05 [-.26, .16] | .16 | -.07 [-.28, .14] | .17 | -.08 [-.28, .12] | .18 |
| Contaminant process | -.09 [-.28, .12] | .19 | -.09 [-.30, .12] | .19 | -.12 [-.32, .09] | .24 | -.11 [-.33, .11] | .24 | -.15 [-.37, .07] | .37 | -.07 [-.28, .14] | .17 |
| Partial (age and PIQ) | -.05 [-.25, .15] | .15 | -.05 [-.25, .16] | .15 | -.26 [-.44, .06] | 3.10 | -.08 [-.29, .14] | .18 | -.01 [-.23, .20] | .14 | -.08 [-.27, .12] | .18 |

*Note. v* = drift rate, *a* = boundary separation, *ter* = non-decision time. BF = Bayes factor. *The posterior mean correlation and 95% credible intervals are presented for each correlation between ADHD subscale (inattention, hyperactivity/impulsivity) score and diffusion model parameter (*v*, *a*, *ter*). The range of Gelman-Rubin statistics is provided for the parameters of interest (i.e., covariates).

**Supplementary Table 8.**

*Correlations between total hyperactivity/impulsivity and inattention scores and diffusion model parameters in each group for Study 2.*

|  | *v* | | *a* | | *ter* | | *v.SAT* | | *a.SAT* | | *ter.SAT* | |
| --- | --- | --- | --- | --- | --- | --- | --- | --- | --- | --- | --- | --- |
|  | Correlation* | BF | Correlation* | BF | Correlation | BF | Correlation | BF | Correlation* | BF | Correlation* | BF |
| *Hyperactivity/impulsivity* | | | | | | | | | | | | |
| Non-autistic group | -.05 [-.36, .29] | .25 | -.10 [-.43, .26] | .27 | -.21 [-.48, .11] | .48 | -.11 [-.44, .25] | .30 | -.18 [-.48, .16] | .39 | -.23 [-.51, .10] | .62 |
| Autistic group | -.14 [-.37, .10] | .30 | .09 [-.15, .32] | .21 | -.26 [-.48, -.01] | 1.35 | .01 [-.26, .27] | .18 | -.13 [-.39, .14] | .28 | -.09 [-.33, .17] | .22 |
| *Inattention* | | | | | | | | | | | | |
| Non-autistic group | -.04 [-.39, .31] | .26 | -.21 [-.53, .19] | .51 | -.26 [-.53, .08] | .77 | .13 [-.26, .46] | .34 | -.04 [-.38, .33] | .25 | -.20 [-.49, .17] | .47 |
| Autistic group | -.09 [-.32, .15] | .20 | .02 [-.22, .26] | .16 | -.20 [-.42, .05] | .56 | -.13 [-.37, .13] | .28 | -.08 [-.34, .18] | .21 | -.02 [-.27, .22] | .17 |

*Note. v* = drift rate, *a* = boundary separation, *ter* = non-decision time. BF = Bayes factor. *The posterior mean correlation and 95% credible intervals are presented for each correlation between ADHD subscale (inattention, hyperactivity/impulsivity) score and diffusion model parameter (*v*, *a*, *ter*). The range of Gelman-Rubin statistics is provided for the parameters of interest (i.e., covariates).

**Supplementary Table 9.**

*Correlations between diffusion model parameters and DCDQ, sensory processing, and TOWRE scores across the whole sample in basic joint models for Study 2.*

| Covariate | *v* | | *a* | | *ter* | | *v.SAT* | | *a.SAT* | | *ter.SAT* | |
| --- | --- | --- | --- | --- | --- | --- | --- | --- | --- | --- | --- | --- |
|  | Correlation* | BF | Correlation* | BF | Correlation | BF | Correlation | BF | Correlation* | BF | Correlation* | BF |
| DCDQ | .08 [-.12, .28] | .18 | .06 [-.14, .26] | .16 | .10 [-.11, .29] | .21 | -.04 [-.25, .17] | .15 | .12 [-.10, .32] | .24 | .14 [-.06, .34] | .33 |
| Sensory OR | .01 [-.18, .21] | .13 | .13 [-.07, .32] | .29 | -.11 [-.30, .09] | .24 | .00 [-.22, .21] | .14 | .07 [-.14, .28] | .17 | -.01 [-.21, .19] | .13 |
| Sensory UR | .00 [-.20, .20] | .13 | -.18 [-.37, .02] | .57 | -.20 [-.38, .00] | .86 | -.03 [-.24, .19] | .15 | -.12 [-.33, .09] | .27 | -.14 [-.33, .06] | .33 |
| TOWRE SWE | .02 [-.18, .22] | .13 | -.27 [-.45, -.07] | 4.36 | -.24 [-.42, -.04] | 1.98 | .03 [-.19, .23] | .14 | -,13 [-.33, .09] | .30 | -.15 [-.35, .05] | .40 |
| TOWRE PDE | -.02 [-.22, .18] | .14 | -.18 [-.37, .02] | .62 | -.05 [-.25, .15] | .15 | .12 [-.09, .33] | .27 | -.15 [-.35, .06] | .37 | -.07 [-.27, .13] | .17 |

**Supplementary Table 10.**

*Correlations between diffusion model parameters and DCDQ, sensory processing, and TOWRE scores across the whole sample in basic joint models for Study 2, while controlling for age and PIQ.*

| Covariate | *v* | | *a* | | *ter* | | *v.SAT* | | *a.SAT* | | *ter.SAT* | |
| --- | --- | --- | --- | --- | --- | --- | --- | --- | --- | --- | --- | --- |
|  | Correlation* | BF | Correlation* | BF | Correlation | BF | Correlation | BF | Correlation* | BF | Correlation* | BF |
| DCDQ | .07 [-.14, .27] | .16 | .06 [-.15, .26] | .16 | .11 [-.09, .31] | .23 | -.01 [-.23, .20] | .14 | .07 [-.15, .28] | .18 | .15 [-.06, .34] | .36 |
| Sensory OR | .00 [-.20, .20] | .13 | .13 [-.07, .33] | .29 | -.08 [-.27, .12] | .17 | -.02 [-.23, .20] | .15 | .09 [-.13, .30] | .20 | -.02 [-.22, .18] | .13 |
| Sensory UR | .02 [-.18, .22] | .13 | -.17 [-.37, .03] | .50 | -.23 [-.41, -.04] | 1.81 | -.05 [-.26, .17] | .16 | -.09 [-.30, .13] | .20 | -.15 [-.34, .06] | .34 |
| TOWRE SWE | .05 [-.15, .25] | .16 | -.27 [-.45, -.07] | 5.15 | -.27 [-.45, -.08] | 5.49 | .04 [-.18, .25] | .15 | -.13 [-.34, .09] | .28 | -.14 [-.34, .06] | .33 |
| TOWRE PDE | -.03 [-.23, .17] | .14 | -.20 [-.39, .01] | .83 | -.09 [-.28, .11] | .19 | .28 [-.08, .34] | .13 | -.19 [-.39, .02] | .64 | -.06 [-.25, .15] | .16 |
